# Switch From Pauli‐Lowering to LUMO‐Lowering Catalysis in Brønsted Acid‐Catalyzed Aza‐Diels‐Alder Reactions

**DOI:** 10.1002/open.202100172

**Published:** 2021-08-05

**Authors:** Song Yu, F. Matthias Bickelhaupt, Trevor A. Hamlin

**Affiliations:** ^1^ Department of Theoretical Chemistry Amsterdam Institute of Molecular and Life Sciences (AIMMS) Amsterdam Center for Multiscale Modeling (ACMM) Vrije Universiteit Amsterdam De Boelelaan 1083 1081 HV Amsterdam The Netherlands) and; ^2^ Institute for Molecules and Materials (IMM) Radboud University Heyendaalseweg 135 6525 AJ Nijmegen The Netherlands

**Keywords:** activation strain models, azadienes, Diels-Alder reactions, density functional calculations, reactivity

## Abstract

Brønsted acid‐catalyzed inverse‐electron demand (IED) aza‐Diels‐Alder reactions between 2‐aza‐dienes and ethylene were studied using quantum chemical calculations. The computed activation energy systematically decreases as the basic sites of the diene progressively become protonated. Our activation strain and Kohn‐Sham molecular orbital analyses traced the origin of this enhanced reactivity to i) “Pauli‐lowering catalysis” for *mono*‐protonated 2‐aza‐dienes due to the induction of an asynchronous, but still concerted, reaction pathway that reduces the Pauli repulsion between the reactants; and ii) “LUMO‐lowering catalysis” for *multi*‐protonated 2‐aza‐dienes due to their highly stabilized LUMO(s) and more concerted synchronous reaction path that facilitates more efficient orbital overlaps in IED interactions. In all, we illustrate how the novel concept of “Pauli‐lowering catalysis” can be overruled by the traditional concept of “LUMO‐lowering catalysis” when the degree of LUMO stabilization is extreme as in the case of *multi*‐protonated 2‐aza‐dienes.

## Graphical abstract

**Switch it up**! Quantum chemical analyses unveil how Brønsted acids catalyze aza‐Diels‐Alder reactions of 2‐aza‐dienes: *mono*‐protonated dienes adopt the recently discovered mechanism of “Pauli‐lowering catalysis”, whereas the *multi*‐protonated dienes switch to the traditional “LUMO‐lowering catalysis”.

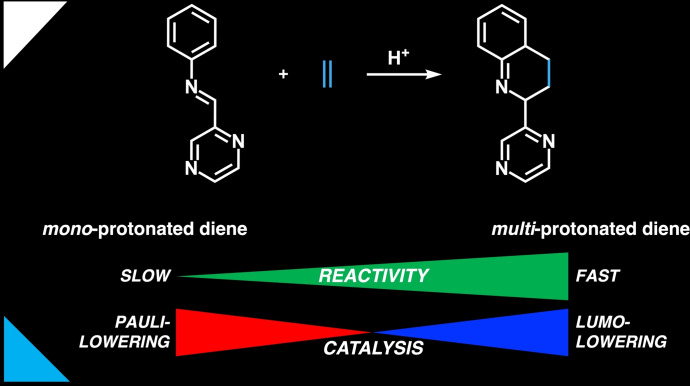

## 1 Introduction

**Scheme 1:**
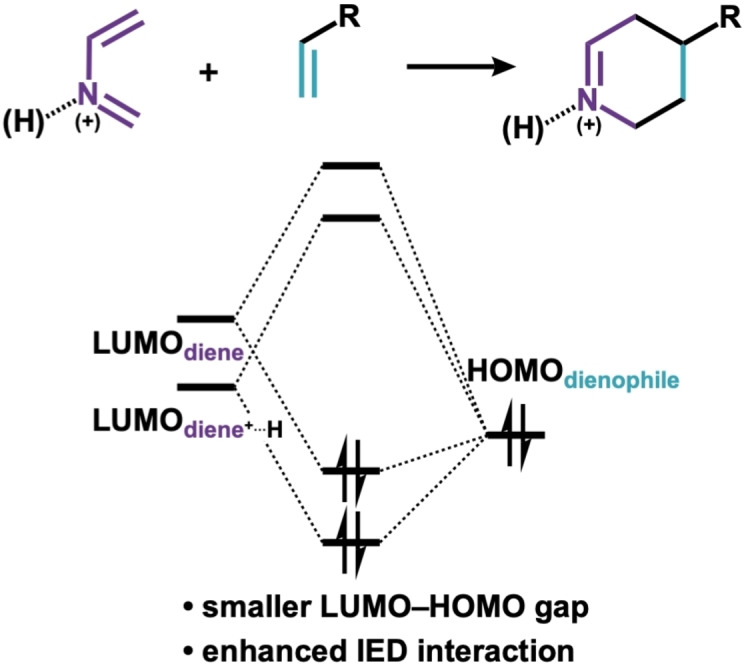
Acid‐catalyzed aza‐Diels‐Alder reactions of 2‐aza‐dienes, with the schematic diagram of the critical donor–acceptor orbital interactions.

Aza‐Diels‐Alder reactions are among the most efficient routes to access heterocycles.^[1]^ The aza‐Diels‐Alder reactions of 2‐aza‐dienes, for instance, furnishes piperidine derivatives that are the common motifs in natural compounds and pharmaceuticals.^[2]^ It is generally understood that the reactivity of 2‐aza‐dienes in Diels‐Alder reactions is governed by the donor‐acceptor interactions between the LUMO_diene_ and the HOMO_dienophile_, i. e., the inverse electron demand (IED) interactions (Scheme 1).^[3]^ These reactions, therefore, are commonly catalyzed by Lewis or Brønsted acids,^[4]^ which upon complexation of the acid to the 2‐aza‐diene induces stabilization of the LUMO_diene_. This “LUMO‐lowering catalysis” concept^[5]^ is thought to lead to a much smaller and more favorable LUMO_diene_–HOMO_dienophile_ energy gap that leads to strongly stabilizing IED orbital interactions (Scheme 1).^[6]^

**Scheme 2:**
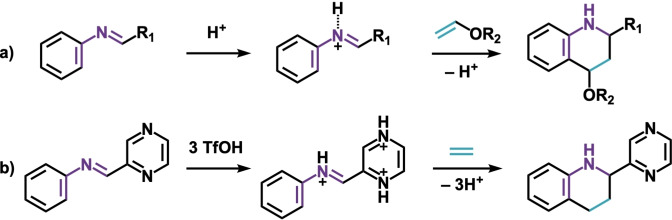
a) Acid‐catalyzed aza‐Diels‐Alder reactions of N‐aryl imines (Povarov reactions); b) the superacid‐catalyzed Povarov reaction.

Besides the parent 2‐aza‐dienes shown in Scheme 1,^[7]^ N‐aryl imines also commonly feature in the acid‐catalyzed aza‐Diels‐Alder reactions.^[8]^ The Povarov reaction, a Diels‐Alder cycloaddition of N‐aryl imines catalyzed by the acids (Scheme 2a)^[9]^ provides convenient access to densely functionalized quinoline derivatives,^[10]^ which are the key blocks in various bioactive compounds.^[11]^ Typically, this transformation is limited to electron‐rich alkenes, such as ethyl vinyl ether and ethyl vinyl sulfide.^[8c]^ Recently, Klumpp and coworkers disclosed the Brønsted superacid, CF_3_SO_3_H, catalyzed aza‐Diels‐Alder between the N‐aryl imines containing multiple basic sites and ethylene.^[12]^ The *multi*‐protonated N‐aryl imine “superelectrophiles”^[13]^ were expected to exhibit highly stabilized LUMOs that could be the origin of the enhanced Diels‐Alder reactivity (Scheme 2b).[^12^, ^13^]

**Scheme 3:**
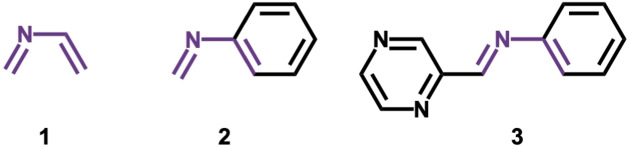
2‐Aza‐dienes studied in this study.

As described above, the acid‐catalyzed aza‐Diels‐Alder reaction of various 2‐aza‐dienes are employed in organic synthesis, but the “LUMO‐lowering catalysis” mechanism^[5]^ has solely been attributed to the enhanced donor–acceptor interactions caused by the stabilized LUMOs of cationic 2‐aza‐dienes upon protonation.[^6^, ^12^] Our previous studies of Lewis acid‐catalyzed Diels‐Alder reactions revealed that Lewis acids activate dienophiles by reducing the Pauli repulsion between the reactants and not due to the previously expected enhanced donor–acceptor interactions.[^14^, ^15^] In the present study, we aimed to uncover the actual mechanism of Brønsted acid‐catalyzed inverse electron demand aza‐Diels‐Alder reactions of 2‐aza‐dienes using density functional theory (DFT) calculations at BP86/TZ2P as implemented in ADF.[^14^, ^16^] Three representative 2‐aza‐dienes were investigated (Scheme 3): the parent 2‐aza‐diene **1**,^[7]^ the archetypal N‐aryl imine used in Povarov reactions **2**,[^8^, ^9^] and the N‐aryl imine containing multiple protonation sites **3**.^[10]^ Ethylene was chosen as the dienophile and proton (**H**)^[7d.e,9,12]^ was selected as the Brønsted acid. The activation strain model (ASM) of reactivity^[17]^ in combination with the matching canonical energy decomposition analysis (EDA)^[18]^ were employed to elucidate the physical factors controlling the Diels‐Alder reactivity of 2‐aza‐dienes.

## 2 Results and Discussion

### 2.1 Brønsted Acid‐Catalyzed Reactions

**Figure 1:**
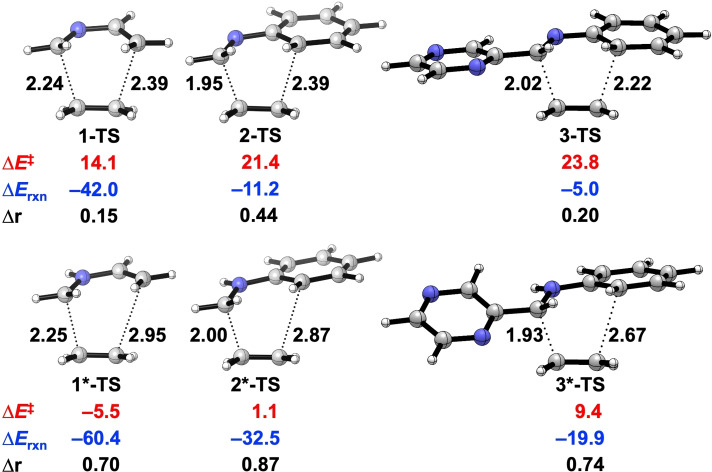
Transition state structures with newly forming bond lengths (Å), activation energies (Δ*E* ^≠^, kcal mol^−1^), reaction energies (Δ*E*_rxn_, kcal mol^−1^), and length differences between newly forming bonds (Δr, Å), for aza‐Diels‐Alder reactions between 2‐aza‐dienes (**1**–**3** and 1*–3*) and ethylene. Note that 3* is the most stable tautomer among others (see Figure S1). All data were computed at BP86/TZ2P.

First, the catalytic effect of Brønsted acids in aza‐Diels‐Alder reactions was studied by comparing the reactivity of the archetypal 2‐aza‐dienes **1–3** and the corresponding protonated 2‐aza‐diene 1*–3*, which are proposed intermediates of Brønsted acid‐catalyzed aza‐Diels‐Alder reactions.[^7d^, ^7e^, ^9^, ^12^] Figure 1 shows the transition state structures and computed activation and reaction energies of the aza‐Diels‐Alder reactions between 2‐aza‐dienes (**1**–**3** and 1*–3*) and ethylene. It is evident that the catalyzed reactions, that is, the reactions of protonated 2‐aza‐dienes (1*–3*) go with significantly lowered activation barriers and more favorable reaction energies compared to their uncatalyzed counterparts. These computed trends in reactivity at BP86/TZ2P^[19]^ agree well with those calculated with an explicit dispersion correction (BP86‐D3(BJ)/TZ2P//BP86/TZ2P)^[20]^ and M06‐2X/TZ2P//BP86/TZ2P,^[21]^ as well as when solvent effects are included at COSMO(DCM)BP86/TZ2P/BP86/TZ2P^[22]^ (see Table S1). Moreover, an inspection of the transition‐state (TS) geometries reveals that the aza‐Diels‐Alder reactions of protonated 2‐aza‐dienes are much more asynchronous than the original reactions: the length differences between two newly forming bonds (Δr) become more pronounced (Figure 1). The differing degree of asynchronicity and its role in Lewis acid‐catalyzed Diels‐Alder reactions has previously been highlighted by us.^[14]^

**Figure 2:**
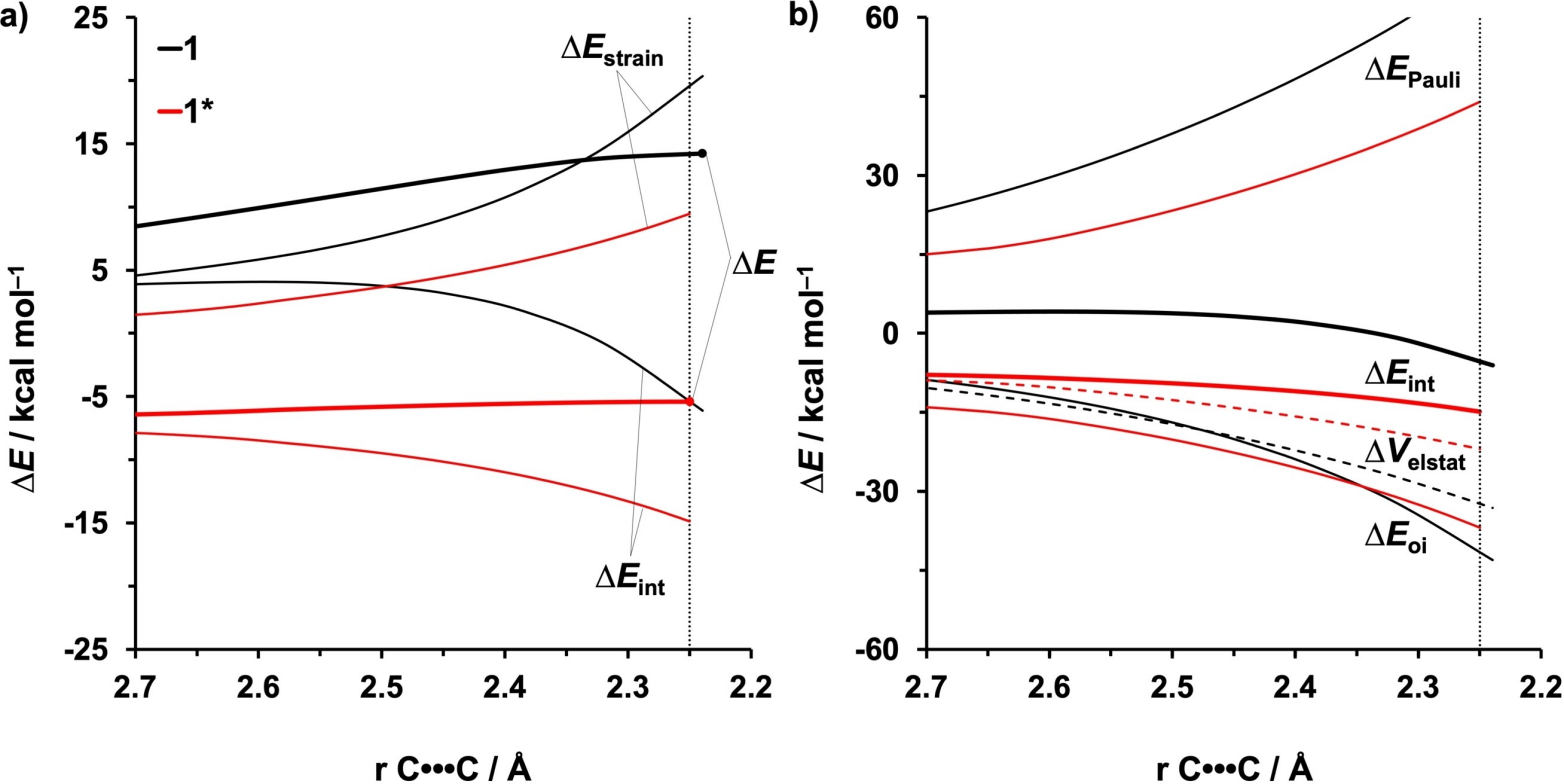
a) Activation strain and b) energy decomposition analyses of aza‐Diels‐Alder reactions between **1**/1* and ethylene along the intrinsic reaction coordinate projected onto the length of the shorter of the two newly forming C⋅⋅⋅C bond, computed at BP86/TZ2P. The vertical dotted line indicates the consistent point where the distance of the shorter forming bond is 2.25 Å.

To probe the origin of the enhanced reactivity and the increased asynchronicity of the aza‐Diels‐Alder reactions of protonated 2‐aza‐dienes, we turned to the activation strain model (ASM). The electronic energy (Δ*E*) is decomposed into two terms: the strain energy (Δ*E*_strain_) that results from the distortion of the individual reactants and the interaction energy (Δ*E*_int_) between the deformed reactants along the reaction coordinate.^[17]^ In this study, all energy terms were projected onto the length of the shorter one of the two forming C⋅⋅⋅C bonds, which undergoes a well‐defined change during the reaction and has proven to provide reliable results for Diels‐Alder reactions.[^14^, ^15^, ^16^, ^23^] In the following, we compare the reactivity of **1** and 1* (Figure 2) and also find that the same general conclusions hold for the other systems, that is, **2**/2* and **3**/3* (Figures S6–S7). Analysis of Figure 2a reveals that the aza‐Diels‐Alder reaction of 1* (red) goes with a lower activation barrier than the reaction of **1** (black), due to the combined effect of a less destabilizing Δ*E*_strain_ and a much more stabilizing Δ*E*_int_.

**Figure 3:**
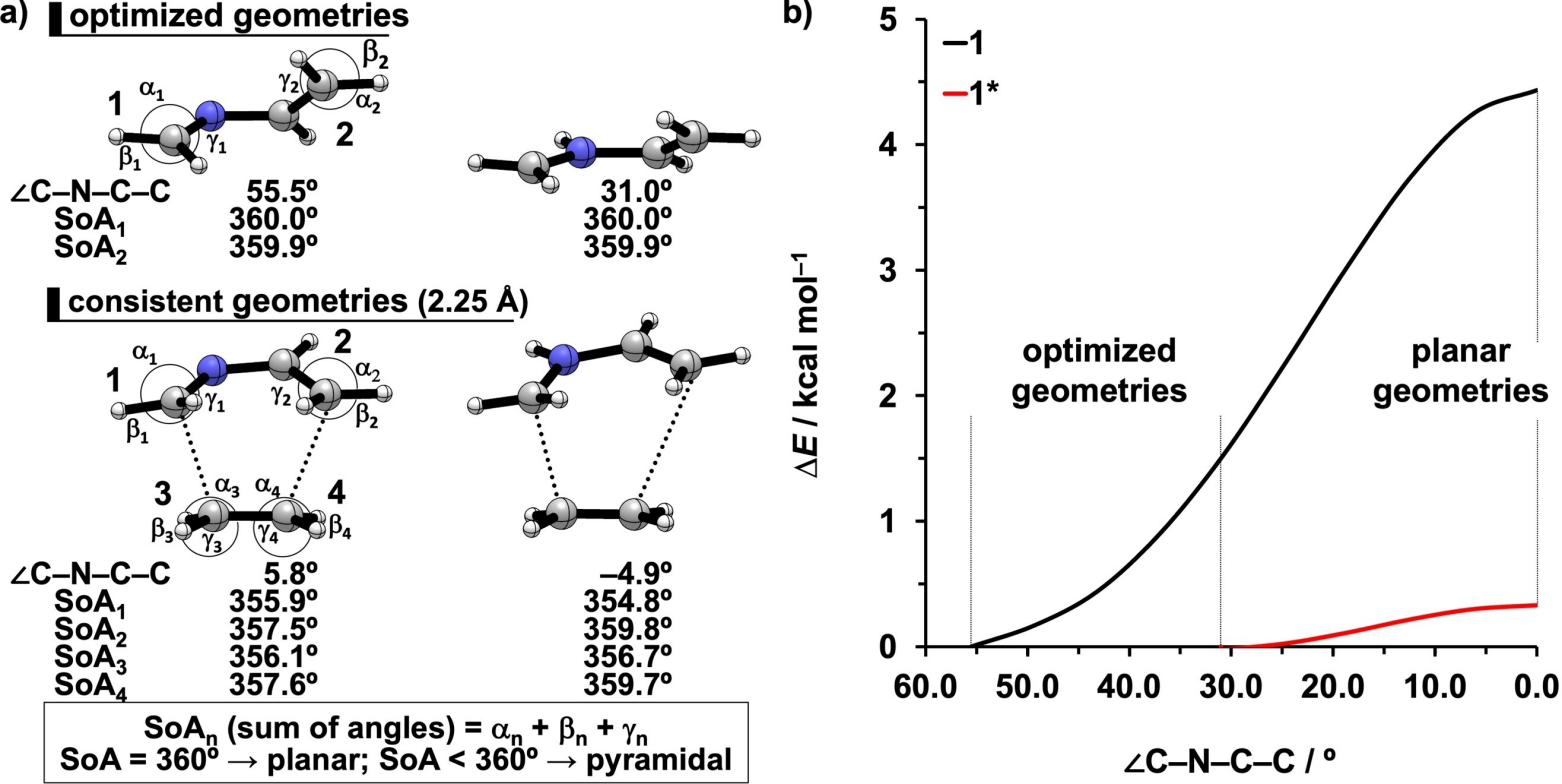
a) Optimized geometries and consistent geometries (shorter forming C⋅⋅⋅C bond is 2.25 Å) of **1** and 1*, including the geometric information in structures; b) computed relative energies of **1** and 1* optimized at various constrained dihedral angles of backbone. All data were computed at BP86/TZ2P.

The less destabilizing Δ*E*_strain_ associated with the aza‐Diels‐Alder reaction of 1* originates from a combined effect of the more asynchronous reaction mode and the pre‐distorted structure of the diene (Figure S2). From our previous work, we know that the more asynchronous Diels‐Alder reaction goes with the formation of the first C−C bond ahead of the other C−C bond, causing the involved terminal carbons to pyramidalize sequentially instead of simultaneously, which goes with less deformation along the reaction around the TS (even though in the eventual product of the addition, the strain is identical for the synchronous and asynchronous reactions).[^14^, ^24^] In this case, we again see that, around the TS region of the process, the more asynchronous reaction 1* goes with less pyramidalization of the terminal carbons, as compared with the reaction **1** (Figure 3a): at the consistent geometries (the distance of the shorter forming bond is 2.25 Å), the SoAs (sum of angles around the atom)^[24]^ of the terminal carbons at the longer forming bond (i. e., SoA_2_ and SoA_4_) are less than 360° (slightly pyramidal) for **1** but nearly exactly 360° (planar) for 1*. The other contributor to a reduced strain of 1* comes from the lesser distortion of the backbone. The optimized geometry of **1** has a large dihedral angle of the C−N−C−C backbone (55.5°, Figure 3a).[^3a^, ^25^] In order to react with ethylene, the 2‐aza‐diene **1** must adopt an *s*‐*cis* conformation in the transition state where the dihedral angle of the backbone is <10° (Figure 3a) and this change in conformation goes with large destabilizing energy (Figure 3b). The protonated 1*, however, is pre‐distorted by the interaction with the proton, yielding a larger C−N−C angle (**1**: 120.5°; 1*: 128.2°) that maximizes the interaction with the proton (please see Figure S3 and associated text for a more detailed analysis). The increased C−N−C angle reduces the repulsion between the hydrogens on the terminal carbons of the 1*.[^3a^, ^25^] This, in turn, allows for a smaller dihedral angle of the backbone (31.0°, Figure 3a) which is electronically preferred by the conjugated π system. Therefore, only relatively low strain energy is needed for 1* to adopt a planar geometry, as compared with **1** (Figure 3b). These same general conclusions also rationalize the less destabilizing strain energy for reactions of 2* and 3* compared to reactions of **2** and **3**, respectively (Figure S8).

**Figure 4:**
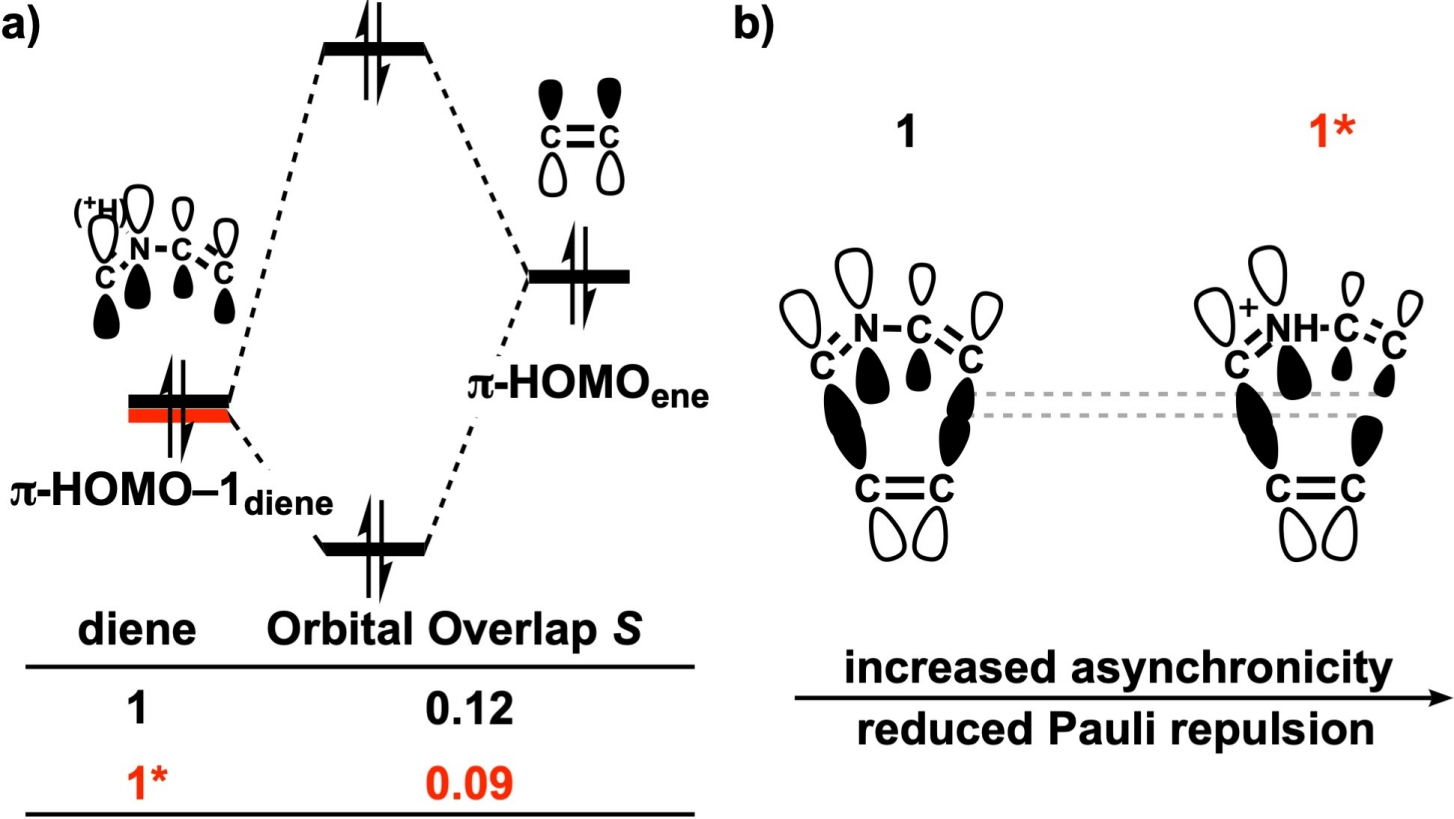
a) Schematic diagrams of the key closed‐shell orbital interactions with overlaps for the aza‐Diels‐Alder reactions between **1**/1* and ethylene, computed at on consistent geometry where shorter forming C⋅⋅⋅C bond is 2.25 Å at BP86/TZ2P; b) the illustration of the closed‐shell orbital interactions.

The even more profound difference in Δ*E*_int_ between the aza‐Diels‐Alder reaction of **1** and 1* was then analyzed by means of the EDA (Figure 2b). Our canonical EDA decomposes the Δ*E*_int_ into three physically meaningful energy terms: classical electrostatic interaction (Δ*V*_elstat_), steric (Pauli) repulsion (Δ*E*_Pauli_) which, in general, arises from the repulsion between the closed‐shell orbitals of both reactants, and stabilizing orbital interaction (Δ*E*_oi_) that accounts, among others, for the HOMO–LUMO interactions.^[18]^ We found that the aza‐Diels‐Alder reaction of 1* benefits predominantly from a less destabilizing Δ*E*_Pauli_ along the entire course of the reaction coordinate, which ultimately leads to the more stabilizing interaction energy when compared with **1**. Interestingly, 1* goes with a less stabilizing Δ*V*_elstat_ and Δ*E*_oi_ at and around the transition state structure seemingly at odds with the “LUMO‐lowering concept”. The difference in Δ*E*_Pauli_ between the reaction of **1** and 1* can be understood by inspecting the critical closed‐shell, two‐orbital four‐electron, orbital interactions between the reactants at the consistent geometries (shorter forming C⋅⋅⋅C bond is 2.25 Å, Figure 4a).^[26]^ The most significant contributor takes place between the π‐HOMO‐1_diene_, i. e., the highest occupied π‐orbital of the diene with no nodal plane (see Figure S4a for the nomenclature of frontier molecular orbitals), and the π‐HOMO_ene_. The aza‐Diels‐Alder reaction of 1* has a smaller overlap of this interaction (0.09) than that of **1** (0.12), which manifests as the less destabilizing Δ*E*_Pauli_ for the reaction of 1*. The reduced overlap between the π‐HOMO‐1_diene_ of 1*, which is the in‐phase π‐orbital of the imine, and π‐HOMO_ene_, the in‐phase π‐orbital, is the result of the increased degree of asynchronicity compared to **1** (Figure 4b). The role of the asynchronicity was further verified by performing a numerical experiment whereby the reaction of 1* was forced to be synchronous: the overlap of this closed‐shell orbital interaction increases from 0.09 to 0.10. The other contributor to the reduced π‐HOMO‐1_diene_–π‐HOMO_ene_ overlap of 1* is the smaller π‐HOMO‐1_diene_ lobe at the C=C bond (Figure 4b). Since the π‐HOMO‐1_diene_ is the bonding combination of the π orbitals of the C=N and C=C bonds and the protonated C=N (i. e., C=NH^+^) has a lowered π orbital, the C=N bond acquires a larger weight in the π‐HOMO‐1_diene_ of 1* at the expense of a reduced contribution from the C=C bond (see Figure S5). Therefore, the π‐HOMO‐1_diene_ of 1* has a smaller orbital lobe at the C=C bond (Figure 4b and Figure S5), which overlaps less efficiently with the π‐HOMO_ene_.

The widely adopted “LUMO‐lowering catalysis”^[5]^ is not the operative activation mechanism for the general Brønsted acid‐catalyzed Diels‐Alder reactions of *mono*‐protonated 2‐aza‐dienes (Figure 2b). Despite the fact that the protonated 1* exhibits a stabilized π*‐LUMO_diene_ and, thus, a smaller and more favorable IED π*‐LUMO_diene_–π‐HOMO_ene_ gap, it goes with a poor orbital overlap (*S*_**1**_=0.20, *S*_1*_=0.18. See Figure S4), due to the combined effect of the smaller amplitude of the π*‐LUMO_diene_ at the C=C bond involved in the longer newly forming bond and the increased asynchronicity. Similar to the π‐HOMO‐1_diene_, the π*‐LUMO_diene_ is the bonding combination of the π* orbitals of the C=N and C=C bonds, the protonated C=N (i. e., C=NH^+^) has a lowered π* orbital that contributes more to the π*‐LUMO_diene_, making the C=C bond contribute less to the π*‐LUMO_diene_ (see Figure S5). Thus, the less efficient IED orbital overlap acts to offset the stabilization‐effect of the IED energy gap and ultimately leads to very similar orbital interactions for **1** and 1* in the TS region of the potential energy surface (Figure 2b).

### 2.2 Brønsted Superacid‐Catalyzed Reactions

**Figure 5:**
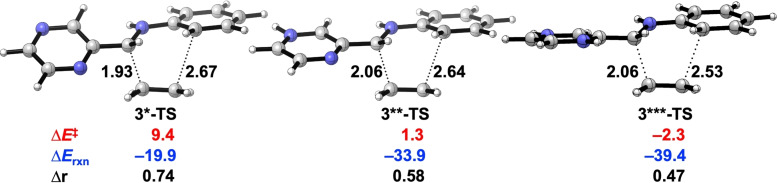
Transition state structures with forming bond lengths (Å), activation energies (Δ*E* ^≠^, kcal mol^−1^), reaction energies (Δ*E*_rxn_, kcal mol^−1^), and length differences between newly forming bonds (Δr, Å) for aza‐Diels‐Alder reactions between protonated N‐aryl imines 3*, 3**, and 3*** with ethylene. Note that 3** is the most stable tautomer among others (Figure S1). All were computed at BP86/TZ2P.

The Nazarov^[27]^ and Povarov reactions^[12]^ are two examples of pericyclic reactions that can also be catalyzed by Brønsted superacids. The highly reactive *multi*‐protonated reactants, which are known as the “superelectrophiles”,^[13]^ are confirmed intermediates that feature in the Brønsted superacid‐catalyzed reactions.^[13c]^ In order to reveal the mechanism of the Brønsted superacid‐catalyzed aza‐Diels‐Alder reactions, the aza‐Diels‐Alder reactivity of the *multi*‐protonated N‐aryl imine **3** ^[12]^ was studied by systematic protonation of the basic N‐sites on the diene. Figure 5 shows the transition state structures for the aza‐Diels‐Alder reactions of the *mono*‐protonated (3*), *di*‐protonated (3**), and *tri*‐protonated (3***) aza‐dienes. Interestingly, we see that the activation and reaction energy become progressively more stabilized and the transition state becomes *less* asynchronous on going from 3* to 3***.

**Figure 6:**
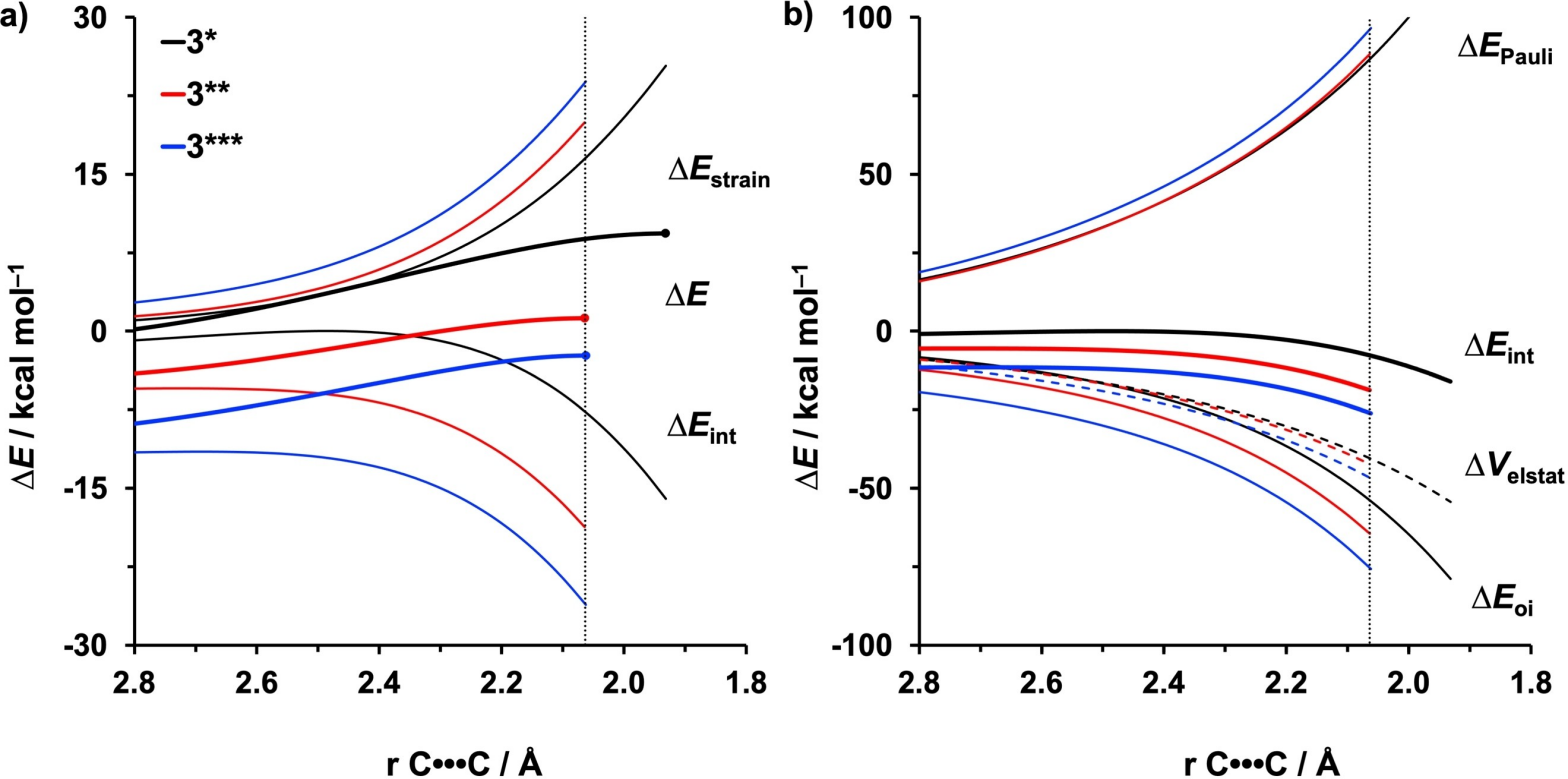
a) Activation strain and b) energy decomposition analyses of aza‐Diels‐Alder reactions between 3*/3**/3*** and ethylene along the intrinsic reaction coordinate projected onto the length of the shorter of the two newly forming C⋅⋅⋅C bond, computed at BP86/TZ2P. The vertical dotted line indicates the consistent point where the distance of the shorter forming C⋅⋅⋅C bond is 2.06 Å.

To pinpoint the origin of the additionally enhanced reactivity of the *multi*‐protonated 3** and 3*** in aza‐Diels‐Alder reactions, we again turned to the ASM. The results shown in Figure 6a reveal that the reaction barrier becomes lower from 3* to 3***, exclusively due to an increasingly more stabilizing Δ*E*_int_. The Δ*E*_strain_ term, in this case, is not responsible for the enhanced reactivity of 3** and 3***. Furthermore, the EDA of Figure 6b shows that the more stabilizing Δ*E*_int_ of 3** and 3*** originates from a more stabilizing Δ*E*_oi_ supported by a slightly more stabilizing Δ*V*_elstat_.

**Figure 7:**
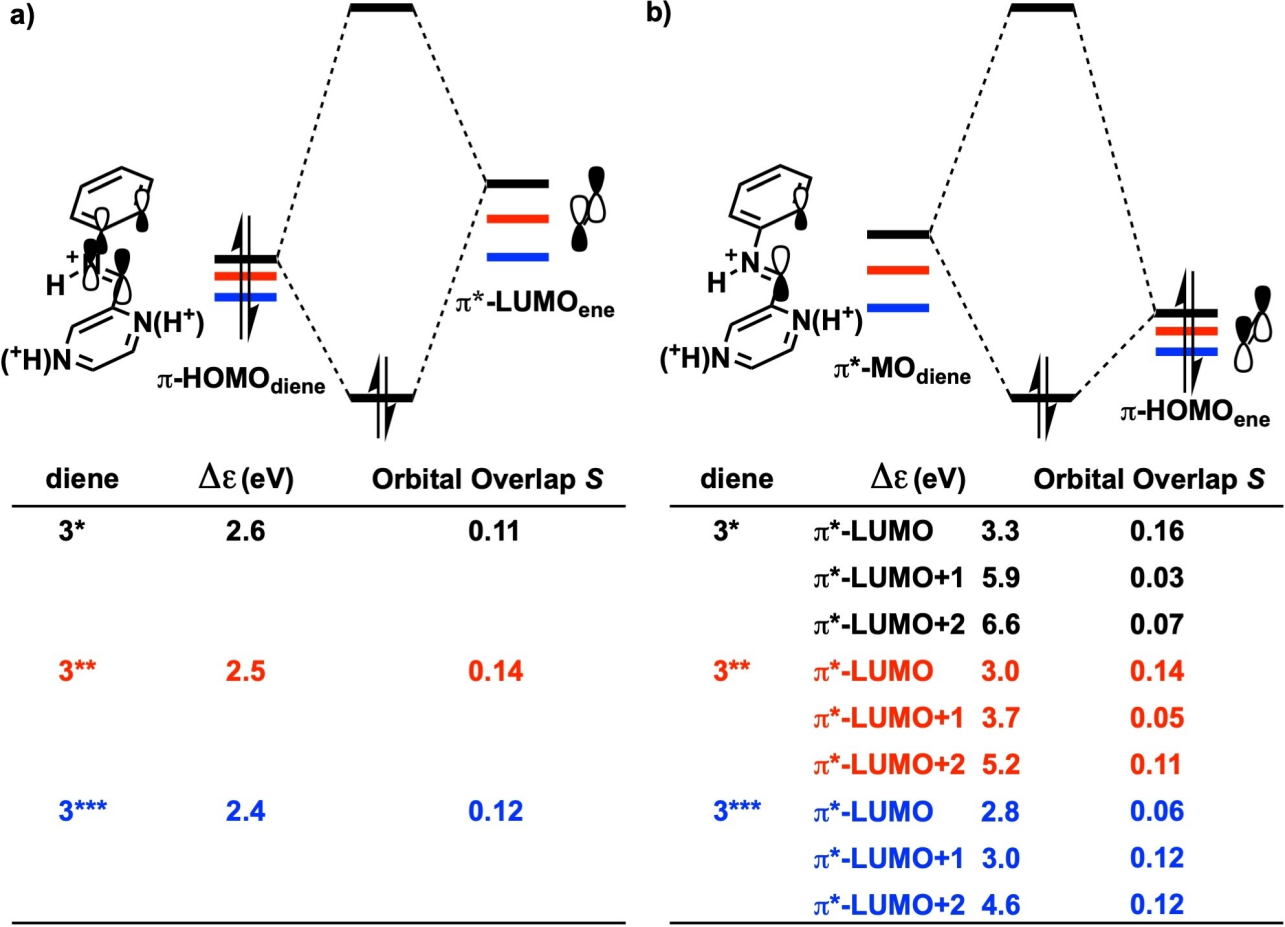
Schematic diagrams of the a) normal electron demand (NED) interactions of the π‐HOMO_diene_–π*‐LUMO_ene_ and b) inverse electron demand (IED) interactions of the π*‐MO_diene_–π‐HOMO_ene_, with the computed gaps Δϵ (in eV) and orbital overlaps *S*, for the aza‐Diels‐Alder reactions of 3*, 3**, and 3*** at the consistent geometries (distance of the shorter forming C⋅⋅⋅C bond is 2.06 Å). All were computed at BP86/TZ2P.

The origin of the more stabilizing Δ*E*_oi_ for the aza‐Diels‐Alder reactions of 3** and 3*** was uncovered by inspecting the critical frontier molecular orbital interactions at the consistent geometries where the distance of the shorter forming C⋅⋅⋅C bond is 2.06 Å. The NED interaction occurs between the π‐HOMO_diene_ and π*‐LUMO_ene_ and becomes moderately stabilized when going from 3* to 3*** (for numerical results see Figure S9a).^[28]^ This slight enhancement in NED interaction on going from 3* to 3*** was also confirmed by NOCV (natural orbitals for chemical valence) analyses (see Figure S9c).^[29]^ We noticed that the π‐HOMO_diene_ becomes increasingly more stabilized as the diene becomes progressively protonated, as does the corresponding π*‐LUMO_ene_ (Figure S10a),^[30]^ which, in turn, leads to the smaller π‐HOMO_diene_–π*‐LUMO_ene_ gaps (Figure 7a) and slightly enhanced NED interactions for the reactions of 3* and 3***. The stabilization of the π*‐LUMO_ene_ upon the protonation of the diene originates from the π*‐LUMO_ene_ being oriented towards, and in close proximity to, the external positive potential of the protonated diene (Figure S11). In addition, we see that the IED interaction is substantially strengthened on going from 3* to 3*** (for numerical results see Figure S9b and NOCV results in Figure S9d), and this is the main source of the stabilized Δ*E*_oi_ and enhanced reactivity of the aza‐Diels‐Alder reactions on going from 3* to 3***. Three π*‐molecular orbitals of the diene (π*‐MO_diene_) were identified to contribute to the IED interactions with the π‐HOMO_ene_: the π*‐LUMO_diene_ and two higher lying virtual orbitals denoted π*‐LUMO+1_diene_ and π*‐LUMO+2_diene_ (see DFT‐computed plots of π*‐MO_diene_ in Figure S12). These π*‐LUMO(s) are all stabilized upon protonation on going from 3* to 3*** (Figure S10b) which causes the IED gaps of π*‐LUMO_diene_−π‐HOMO_ene_, π*‐LUMO+1_diene_−π‐HOMO_ene_, and π*‐LUMO+2_diene_−π‐HOMO_ene_ all to become much smaller (Figure 7b), leading to the significantly enhanced IED interactions for the reactions of 3** and 3***. Therefore, it becomes evident that the *multi*‐protonated 2‐aza‐dienes adopt the “LUMO‐lowering catalysis”.

**Figure 8:**
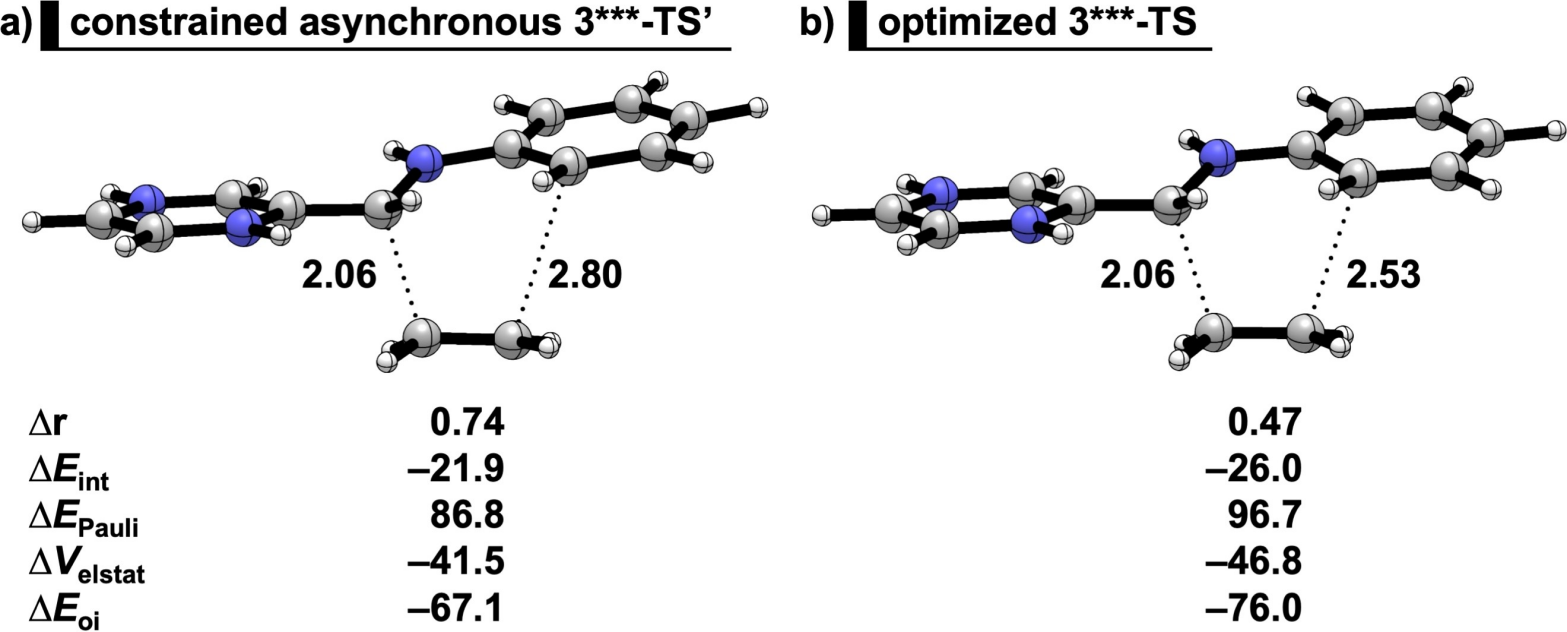
Energy decomposition analyses on the a) constrained asynchronous 3***‐TS’ where the Δr is forced to be 0.74 Å, and b) optimized 3***‐TS where Δr=0.47. All energy terms are in kcal mol^−1^ and computed at BP86/TZ2P.

Our analysis also reveals how the degree of asynchronicity in the transition state of a Diels‐Alder reaction is the result of two counteracting factors: the minimization of the destabilizing Pauli repulsions (asynchronous mode)^[31]^ and the maximization of the stabilizing orbital and electrostatic interactions (synchronous mode). In the previous section, we have already established that the “Pauli‐lowering catalysis”‐controlled reactions have the more asynchronous TS (Figure 1) that minimizes the overlaps of the closed‐shell orbital interactions (Figure 4).[^14^, ^15^] In this case, the superelectrophiles 3** and 3*** display such stabilized LUMO(s) that the “LUMO‐lowering catalysis” becomes operative (Figure 6 and 7). Accordingly, these “LUMO‐lowering catalysis”‐controlled reactions of 3** and 3*** become more synchronous (Figure 5), to maximize the stabilizing orbital and electrostatic interactions. This was verified by a comparison between the optimized 3***‐TS with constrained asynchronous 3***‐TS’ (Figure 8): the 3***‐TS (Δr=0.47 Å) is more synchronous and benefits from additional stabilizing interactions compared to the artificially asynchronous 3***‐TS’ (Δr=0.74 Å). The stabilizing orbital and electrostatic interactions of 3***‐TS overrule the increase in the Pauli repulsion and ultimately lead to a more stabilizing total interaction energy and thus a more favorable transition state structure compared to 3***‐TS’ (Figure 8).

## 3 Conclusions

Our quantum chemical exploration pinpointed the factors that determine the trends in reactivity of Brønsted acid‐catalyzed aza‐Diels‐Alder reactions between various 2‐aza‐dienes and ethylene. Protonation vastly enhances the reactivity of 2‐aza‐dienes in Diels‐Alder reactions with ethylene. *Mono*‐protonated 2‐aza‐dienes operate under the regime of “Pauli‐lowering catalysis” whereas *multi*‐protonated 2‐aza‐dienes operate under the widely‐established regime of “LUMO‐lowering catalysis.”

Our findings based on the activation strain model and Kohn‐Sham molecular orbital theory revealed that the *mono*‐protonation of 2‐aza‐dienes catalyzes the reactions by reducing the strain energy of the diene and Pauli repulsion between the reactants. This is due to that the *mono*‐protonated reaction adopts a more asynchronous reaction mode that demands less deformation of the terminal carbons of the diene and experiences a smaller overlap of the closed‐shell orbital interaction. The expected “LUMO‐lowering catalysis” is not the driving force, because the *mono*‐protonated reaction goes with a poor orbital overlap of the IED interaction that offsets the LUMO‐stabilization effect and ultimately leads to an unvaried IED interaction. On the other hand, the *multi*‐protonation of 2‐aza‐dienes additionally enhances their reactivity by the “LUMO‐lowering catalysis”. In this case, the *multi*‐protonated dienes have such stabilized LUMOs that the LUMO‐lowering effect becomes operative. Moreover, we found that the reactions of *multi*‐protonated 2‐aza‐dienes proceed via a more synchronous pathway which facilitates the orbital overlap of the orbital interactions.

This study shows how “Pauli‐lowering catalysis” can switch to “LUMO‐lowering catalysis” when the degree of LUMO stabilization is extreme as in the case of *multi*‐protonated 2‐aza‐dienes. Furthermore, we establish that Pauli‐lowering catalysis and asynchronous TS structures occur if the reduction in Pauli repulsion is bigger than the loss in stabilizing NED and/or IED HOMO–LUMO interactions whereas LUMO‐lowering catalysis and synchronous TS structures occur if the gain in stabilizing NED and/or IED HOMO–LUMO interactions is bigger than the increase in Pauli repulsion.

## Conflict of interest

The authors declare no conflict of interest.

## Supporting information

As a service to our authors and readers, this journal provides supporting information supplied by the authors. Such materials are peer reviewed and may be re‐organized for online delivery, but are not copy‐edited or typeset. Technical support issues arising from supporting information (other than missing files) should be addressed to the authors.

Supporting Information
