# Supplementary material for: Switch From Pauli‐Lowering to LUMO‐Lowering Catalysis in Brønsted Acid‐Catalyzed Aza‐Diels‐Alder Reactions
Source: ChemistryOpen. 2021 Aug 5;10(8):784–9. doi: 10.1002/open.202100172 (PMC8340067; doi:10.1002/open.202100172)
Supplement: Supplementary file 1 — Supporting Information [file OPEN-10-784-s001.pdf]

ChemistryOpen

Supporting Information

Switch From Pauli-Lowering to LUMO-Lowering Catalysis in Brønsted Acid-Catalyzed Aza-Diels-Alder Reactions

Song Yu, F. Matthias Bickelhaupt,* and Trevor A. Hamlin*

Contents

Computational Methods

Figure S1. Relative electronic energies (kcal mol^{-1}), entropies (kcal mol^{-1}), and Gibbs free energies (kcal mol^{-1}) for different tautomers of **3*** and **3****, computed at BP86/TZ2P.

Table S1. Activation and reaction energies, enthalpies, and Gibbs free energies (in kcal mol^{-1}) for aza-Diels-Alder reactions between 2-aza-dienes and ethylene, computed at BP86/TZ2P, BP86-D3(BJ)/TZ2P, M06-2X/TZ2P and COSMO(DCM)BP86/TZ2P, using BP86/TZ2P geometries.

Figure S2. Decomposition of the strain energies in aza-Diels-Alder reactions between **1**/**1*** and ethylene along the IRC projected on the newly forming C•••C bond, computed at BP86/TZ2P.

Figure S3. Analyses of the protonated 2-aza-diene and imine. All were computed at BP86/TZ2P.

Figure S4. a) Scheme of frontier molecular orbitals (FMO) involved in the Diels-Alder reactions. Diagrams with energy gaps and overlaps of a) the NED π-$HOMO_{diene}$–π*-$LUMO_{ene}$ and b) IED π*-$LUMO_{diene}$–π-$HOMO_{ene}$ interactions for aza-Diels-Alder reactions of **1** and **1*** with ethylene at the consistent geometry (new C•••C bond of 2.25 Å), computed at BP86/TZ2P.

Figure S5. Schematic formation of the FMOs of **1** and **1***, including the contributions (blue) and coefficients (red) of the constituting fragment MOs, with schematic and DFT-computed plots of the MOs.

Figure S6. a) Activation strain and b) energy decomposition analyses of aza-Diels-Alder reactions of **2** and **2*** with ethylene, projected onto the length of the shorter newly forming C•••C bond, computed at BP86/TZ2P.

Figure S7. a) Activation strain and b) energy decomposition analyses of aza-Diels-Alder reactions between **3** and **3*** with ethylene along the IRC projected onto the length of the shorter newly forming C•••C bond, computed at BP86/TZ2P.

Figure S8. a) Optimized and consistent (2.00 Å) geometries of **2** and **2***, and b) optimized and consistent (2.02 Å) geometries of **3** and **3***, including the geometric information within structures.

Figure S9. Computed energies for the a) normal electron demand (NED) interactions between the filled orbitals of diene with the π*-$LUMO_{ene}$ and b) inverse electron demand (IED) interactions between the π*-MO_{diene} with the filled orbitals of ethylene, for the aza-Diels-Alder reactions of **3***, **3****, and **3*****. NOCV deformation densities $\Delta\rho$ (isovalue = 0.002 au; electron flow: red → blue) and associated energies $\Delta E(\rho)$ (in kcal mol^{-1}) for the c) NED and d) IED interactions of the aza-Diels-Alder reactions of **3***, **3****, and **3*****. All were computed at the consistent geometries (2.06 Å) at BP86/TZ2P.

Figure S10. Schematic diagrams of the a) normal electron demand (NED) interactions between the π-$HOMO_{diene}$ and π*-$LUMO_{ene}$ and b) inverse electron demand (IED) interactions between the π*-MO_{diene} and π-$HOMO_{ene}$, with the computed orbital energies ε (in eV) and energy gaps Δε (in eV), for the aza-Diels-Alder reactions of **3***, **3****, and **3*****. All data were computed at the consistent geometries (2.06 Å) at BP86/TZ2P.

Figure S11. Electrostatic potential maps (at 0.06 $Bohr^{-3}$) from 0 (red) to 1 (blue) $Hartree^{-1}$ of the fragmental **3***, **3****, and **3*****, and the DFT-computed plots of the MOs (isovalue = 0.09 $Bohr^{-3/2}$) and energies of the ethylene in proximity to the fragmental **3***, **3****, and **3*****. All data were computed in the consistent geometries (2.06 Å) at BP86/TZ2P.

Figure S12. DFT-computed plots of the MOs (isovalue = 0.03 $Bohr^{-3/2}$) of the diene in the aza-Diels-Alder reactions of **3***, **3****, and **3***** at the consistent geometries (2.06 Å), computed at BP86/TZ2P.

Table S2. Electronic energies, enthalpies, Gibbs free energies, the number and wavelength of the imaginary vibrational frequencies and cartesian coordinates (Å), for all stationary points, computed at BP86/TZ2P using ADF2017.107.

Computational Methods

All calculations were performed with ADF2017.107,[1] using the BP86[2] functional with TZ2P[3] basis set, which has been proven to be accurate in calculating the relative trends in activation and reaction energies for cycloadditions.[4] Frequency calculations were performed to characterize the nature of the stationary points. Local minima presented real frequencies while transition structures had one imaginary frequency associated with the transition vector. All structures were visualized using CYLview.[5] The potential energy surface (PES) was calculated using the intrinsic reaction coordinate (IRC) method,[6] which follows the transition vector (i.e., the vibrational normal mode associated with the reaction and with a negative force constant) from the transition structure towards the reactants and product and was analyzed with the aid of the PyFrag 2019 program.[7] Quantitative analyses of the activation barriers associated with the studied reactions were obtained by means of the activation strain model (ASM) of reactivity.[8] The PES, $\Delta E(\zeta)$, is decomposed into the strain energy, $\Delta E_{\text{strain}}(\zeta)$, and interaction energy, $\Delta E_{\text{int}}(\zeta)$ [Eq. (1)]. All energy terms were

---

[1] a) G. te Velde, F. M. Bickelhaupt, E. J. Baerends, C. Fonseca Guerra, S. J. A. van Gisbergen, J. G. Snijders, T. Ziegler, *J. Comput. Chem.* **2001**, *22*, 931–967; b) C. Fonseca Guerra, J. G. Snijders, G. te Velde, E. J. Baerends, *Theor. Chem. Acc.* **1998**, *99*, 391–403; c) ADF, SCM, Theoretical Chemistry; Vrije Universiteit, Amsterdam, The Netherlands, **2017**; http://www.scm.com.

[2] a) A. D. Becke, *Phys. Rev. A* **1988**, *38*, 3098–3100; b) J. P. Perdew, *Phys. Rev. B* **1986**, *33*, 8822–8824.

[3] E. van Lenthe, E. J. Baerends, *J. Comput. Chem.* **2003**, *24*, 1142–1156.

[4] a) P. Vermeeren, T. A. Hamlin, I. Fernández, F. M. Bickelhaupt, *Angew. Chem.* **2020**, *132*, 6260–6265; *Angew. Chem. Int. Ed.* **2020**, *59*, 6201–6206; b) T. A. Hamlin, D. Svatunek, S. Yu, L. Ridder, I. Infante, L. Visscher, F. M. Bickelhaupt, *Eur. J. Org. Chem.* **2019**, 378–386; c) S. Yu, P. Vermeeren, K. van Dommelen, F. M. Bickelhaupt, T. A. Hamlin, *Chem. Eur. J.* **2020**, *26*,11529–11539; d) S. Yu, P. Vermeeren, T. A. Hamlin, F. M. Bickelhaupt, *Chem. Eur. J.* **2021**, *27*, 5683–5693.

[5] C. Y. Legault, CYLview, 1.0b; Université de Sherbrooke, Sherbrooke, QC, Canada, **2009**; http://www.cylview.org.

[6] a) L. Deng, T. Ziegler, L. Fan, *J. Chem. Phys.* **1993**, *99*, 3823–3835; b) L. Deng, T. Ziegler, *Int. J. Quantum Chem.* **1994**, *52*, 731–765.

[7] a) W.-J. van Zeist, C. Fonseca Guerra, F. M. Bickelhaupt, *J. Comput. Chem.* **2008**, *29*, 312–315; b) X. Sun, T. M. Soini, J. Poater, T. A. Hamlin, F. M. Bickelhaupt, *J. Comput. Chem.* **2019**, *40*, 2227–2233; c) PyFrag 2019: X. Sun, T. Soini, L. P. Wolters, W.-J. van Zeist, C. Fonseca Guerra, T. A. Hamlin, F. M. Bickelhaupt, Vrije Universiteit Amsterdam, The Netherlands.

[8] a) D. H. Ess, K. N. Houk, *J. Am. Chem. Soc.* **2008**, *130*, 10187–10198; b) W.-J. van Zeist, F. M. Bickelhaupt, *Org. Biomol. Chem.* **2010**, *8*, 3118–3127; c) I. Fernández, F. M. Bickelhaupt, *Chem. Soc. Rev.* **2014**, *43*, 4953–4967; d) L. P. Wolters, F. M. Bickelhaupt, *Wiley Interdiscip. Rev. Comput. Mol. Sci.* **2015**, *5*, 324–343; e) F. M. Bickelhaupt, K. N. Houk, *Angew. Chem. Int. Ed.* **2017**, *56*, 10070–10086; f) I F. M. Bickelhaupt, K. N. Houk, *Angew. Chem.* **2017**, *129*, 10204–10221; g) P. Vermeeren, S. C. C. van der Lubbe, C. Fonseca Guerra, F. M. Bickelhaupt, T. A. Hamlin, *Nat. Protoc.* **2020**, *15*, 649–667; h) P. Vermeeren, T. A. Hamlin, F. M. Bickelhaupt, *Chem. Commun.* **2021**, *57*, https://doi.org/10.1039/D1CC02042K.

projected onto the length of the shorter one of the two forming C•••C bonds, which undergoes a well-defined change during the course of the reaction and has proven to provide reliable results for Diels-Alder reactions.[4, 9]

$$\Delta E(\zeta) = \Delta E_{\text{strain}}(\zeta) + \Delta E_{\text{int}}(\zeta) \quad (1)$$

The $\Delta E_{\text{int}}(\zeta)$ is related to the electronic structure of the reactants and their spatial orientation and takes the mutual interactions between the deformed reactants into account. To obtain a deeper insight into the physical mechanism behind $\Delta E_{\text{int}}(\zeta)$, we employed our canonical energy decomposition analysis (EDA),[10] which decomposes the interaction energy between the deformed reactants, within the framework of Kohn-Sham DFT, into three physically meaningful terms [Eq. (2)].

$$\Delta E_{\text{int}}(\zeta) = \Delta V_{\text{elstat}}(\zeta) + \Delta E_{\text{Pauli}}(\zeta) + \Delta E_{\text{oi}}(\zeta) \quad (2)$$

The electrostatic interaction, $\Delta V_{\text{elstat}}(\zeta)$, corresponds to the classical electrostatic interaction between the unperturbed charge distributions of deformed reactants. The Pauli repulsion, $\Delta E_{\text{Pauli}}(\zeta)$, comprises the repulsion between closed-shell orbitals and is, therefore, destabilizing. The orbital interaction, $\Delta E_{\text{oi}}(\zeta)$, accounts for the stabilizing orbital interactions, such as, charge transfer, namely, the interactions between the occupied orbitals of one reactant and the unoccupied orbitals of the other reactant, and polarization, that is, the occupied–unoccupied orbital mixing within one reactant due to the presence of the other reactant.

[9] W.-J. van Zeist, A. H. Koers, L. P. Wolters, F. Matthias Bickelhaupt, *J. Chem. Theory Comput.* **2008**, *4*, 920–928.

[10] a) F. M. Bickelhaupt, E. J. Baerends in *Reviews in Computational Chemistry* (Eds: K. B. Lipkowitz, D. B. Boyd), Wiley, Hoboken, **2000**, pp 1–86; b) R. van Meer, O. V. Gritsenko, E. J. Baerends, *J. Chem. Theory Comput.* **2014**, *10*, 4432; c) L. Zhao, M. von Hopffgarten, D. M. Andrada, G. Frenking, *WIREs Comput. Mol. Sci.* **2018**, *8*, e1345; d) T. A. Hamlin, P. Vermeeren, C. Fonseca Guerra, F. M. Bickelhaupt, "Energy decomposition analysis in the context of quantitative molecular orbital theory" in *Complementary Bonding Analysis* (Ed: S. Grabowsky), De Gruyter, Berlin, Boston, **2021**, pp 199–212.

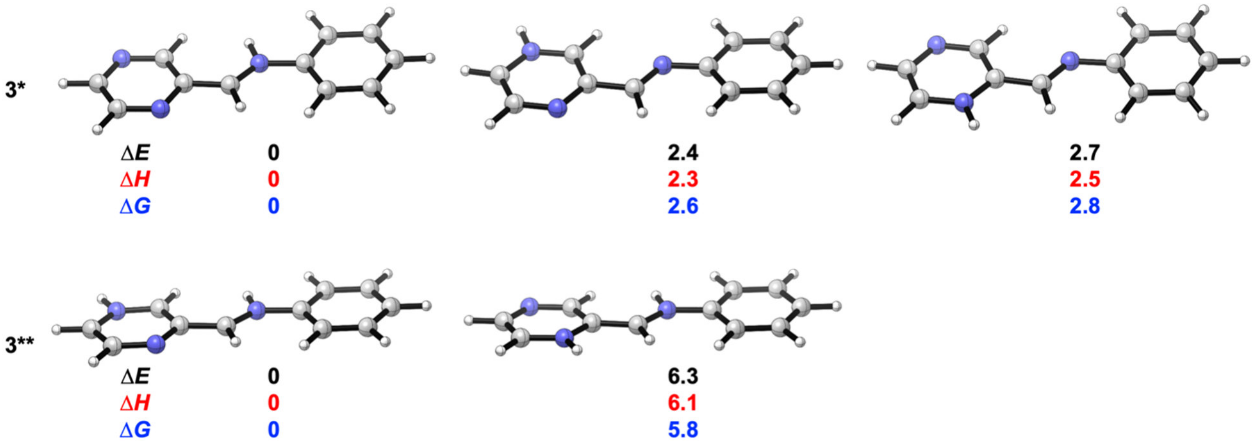

Figure S1. Relative electronic energies (kcal mol^{-1}), entropies (kcal mol^{-1}), and Gibbs free energies (kcal mol^{-1}) for different tautomers of **3*** and **3****, computed at BP86/TZ2P.

Table S1. Activation and reaction energies, enthalpies, and Gibbs free energies (in kcal mol^{-1}) for aza-Diels-Alder reactions between 2-aza-dienes and ethylene, computed at BP86/TZ2P, BP86-D3(BJ)/TZ2P, M06-2X/TZ2P and COSMO(DCM)BP86/TZ2P, using BP86/TZ2P geometries.

			BP86/TZ2P			
	$\Delta E^{\ddagger}$	$\Delta H^{\ddagger}$	$\Delta G^{\ddagger}$	ΔE_{rxn}	ΔH_{rxn}	ΔG_{rxn}
**1**	14.1	15.0	27.2	–42.0	–37.8	–24.3
**1***	–5.5	–4.5	7.0	–60.4	–56.2	–42.5
**2**	21.4	22.4	35.1	–11.2	–7.7	5.6
**2***	1.1	2.1	14.2	–32.5	–29.0	–15.7
**3**	23.8	24.7	37.8	–5.0	–1.9	11.6
**3***	9.4	10.2	23.3	–19.9	–17.3	–1.6
**3****	1.2	2.2	14.6	–33.9	–31.2	–16.1
**3*****	–2.3	–1.6	10.6	–39.4	–36.4	–22.6
			BP86-D3(BJ)/TZ2P//BP86/TZ2P			
	$\Delta E^{\ddagger}$	$\Delta H^{\ddagger}$	$\Delta G^{\ddagger}$	ΔE_{rxn}	ΔH_{rxn}	ΔG_{rxn}
**1**	8.2	9.1	21.4	–46.8	–42.5	–29.1
**1***	–10.8	–9.8	1.7	–65.2	–60.9	–47.3
**2**	14.7	15.7	28.4	–17.2	–13.7	–0.5
**2***	–5.0	–4.0	8.1	–38.7	–35.2	–21.8
**3**	15.3	16.1	29.2	–12.6	–9.5	4.1
**3***	1.4	2.3	15.4	–27.5	–24.9	–9.3
**3****	–7.1	–6.1	6.3	–41.5	–38.7	–23.7
**3*****	–10.7	–10.0	2.3	–46.8	–43.9	–30.1
			M06-2X/TZ2P//BP86/TZ2P			
	$\Delta E^{\ddagger}$	$\Delta H^{\ddagger}$	$\Delta G^{\ddagger}$	ΔE_{rxn}	ΔH_{rxn}	ΔG_{rxn}
**1**	16.6	17.6	29.8	–48.8	–44.5	–31.1
**1***	–4.1	–3.1	8.4	–67.0	–62.8	–49.2
**2**	26.0	27.0	39.7	–16.2	–12.8	0.5
**2***	2.1	3.1	15.1	–39.1	–35.6	–22.3
**3**	25.5	26.3	39.4	–13.2	–10.1	3.4
**3***	8.9	9.7	22.8	–29.6	–26.9	–11.3
**3****	–1.0	0.0	12.4	–47.1	–44.4	–29.4
**3*****	–3.7	–3.0	9.2	–53.6	–50.6	–36.8
			COSMO(DCM)BP86/TZ2P//BP86/TZ2P			
	$\Delta E^{\ddagger}$	$\Delta H^{\ddagger}$	$\Delta G^{\ddagger}$	ΔE_{rxn}	ΔH_{rxn}	ΔG_{rxn}
**1**	13.5	14.4	26.7	–42.4	–38.2	–24.7
**1***	2.5	3.5	15.0	–53.5	–49.3	–35.6
**2**	20.6	21.6	34.2	–11.8	–8.3	4.9
**2***	7.5	8.5	20.6	–25.1	–21.6	–8.2
**3**	23.9	24.7	37.9	–5.3	–2.2	11.3
**3***	14.5	15.4	28.5	–16.7	–14.0	1.6
**3****	11.0	11.9	24.3	–22.1	–19.3	–4.3
**3*****	11.2	11.9	24.1	–23.9	–20.9	–7.1

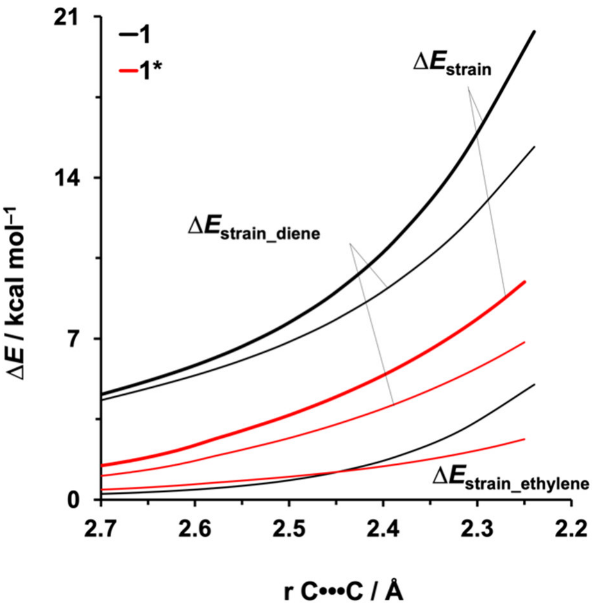

Figure S2. Decomposition of the strain energies in aza-Diels-Alder reactions between **1**/**1*** and ethylene along the IRC projected on the newly forming C•••C bond, computed at BP86/TZ2P.

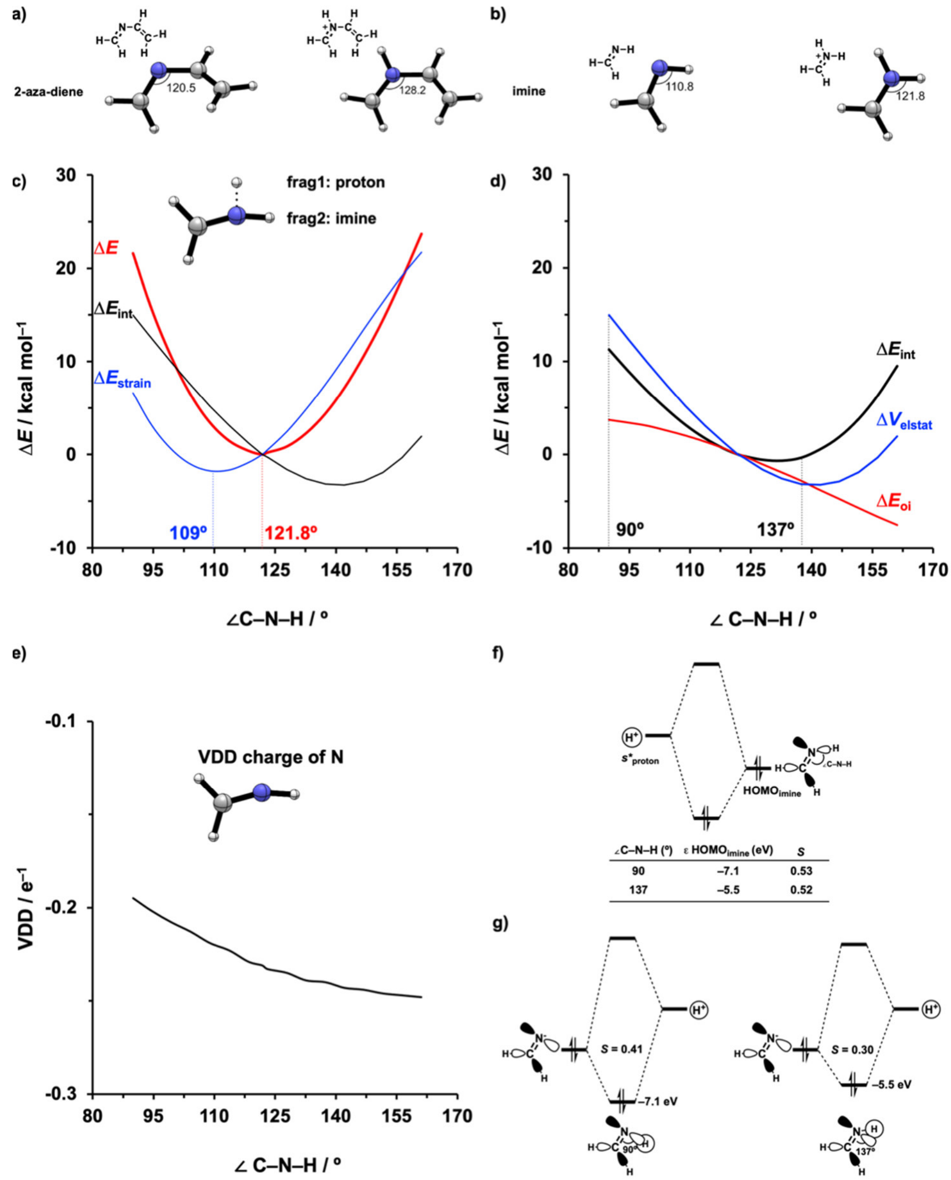

Figure S3. Analyses of the protonated 2-aza-diene and imine computed at BP86/TZ2P.

The C–N–C angle of the 2-aza-diene increases from 120.5º to 128.2º upon protonation (Figure S3a). To rationalize this protonation induced geometric change, we studied an iminium archetypal model system. This model also displays a larger C–N–H angle (121.8º) than the pristine imine (110.8º) (Figure S3b). A relaxed C–N–H scan was performed on the protonated imine followed by an activation strain analysis based on a heterolytic fragmentation involving a proton, H^+, and imine, $H_2C{=}NH$ (Figure S3c). The C–N–H scan reveals a minimum in ΔE, at 121.8º, while the ΔE_{strain}, i.e., the energy of the imine fragment, has a minimum at 109º (Figure S3c). When the C–N–H angle is larger there is a more stabilizing interaction ΔE_{int} between proton and imine. The energy decomposition analysis reveals that the electrostatic and orbital interactions between proton and imine become more stabilizing when the C–N–H angle of imine increases up to 137º (Figure S3d). The stronger electrostatic interaction originates from a more negatively-charged nitrogen of imine with a larger C–N–H angle (Figure S3e). The enhanced orbital interaction is traced back to the higher HOMO of the imine with a larger C–N–H angle that can interact more efficiently with the *s** orbital of proton (Figure S3f). Since the $HOMO_{imine}$ is the bonding mixing of the in-plane orbital of $H_2C{=}N^-$ and the *s** orbital of H^+ (Figure S3g), the imine with the larger C–N–H angle has a less efficient overlap of this interaction that leads to a less stabilizing bonding orbital, i.e., a higher HOMO of imine (Figure S3g). In summary, the protonated imine has a larger C–N–H angle to maximize the stabilizing interaction between proton and the imine.

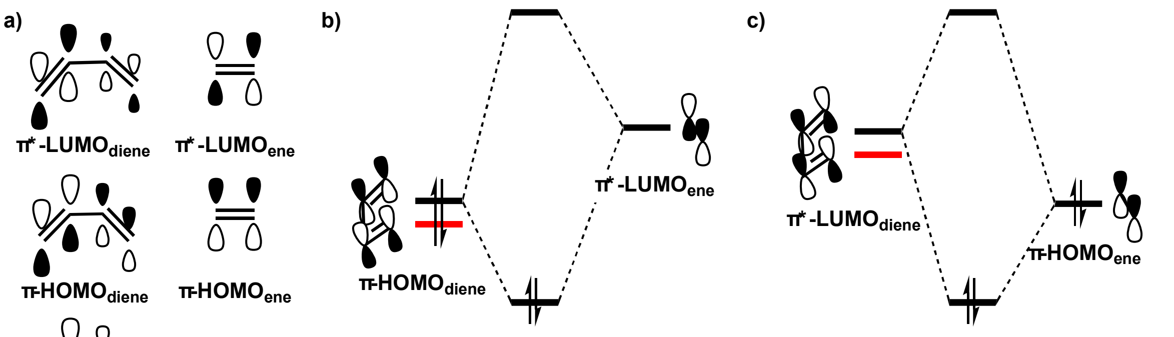

b)

	Δε (eV)	Orbital Overlap *S*
1	2.6	0.18
1*	4.3	0.15

c)

	Δε (eV)	Orbital Overlap *S*
1	3.6	0.20
1*	2.9	0.18

Figure S4. a) Scheme of frontier molecular orbitals (FMO) involved in the Diels-Alder reactions. Diagrams with energy gaps and overlaps of b) the NED π-HOMO$_{diene}$–π*-LUMO$_{ene}$ and c) the IED π*-LUMO$_{diene}$–π-HOMO$_{ene}$ interactions for aza-Diels-Alder reactions of **1** and **1*** with ethylene at the consistent geometry (new C•••C bond of 2.25 Å), computed at BP86/TZ2P.

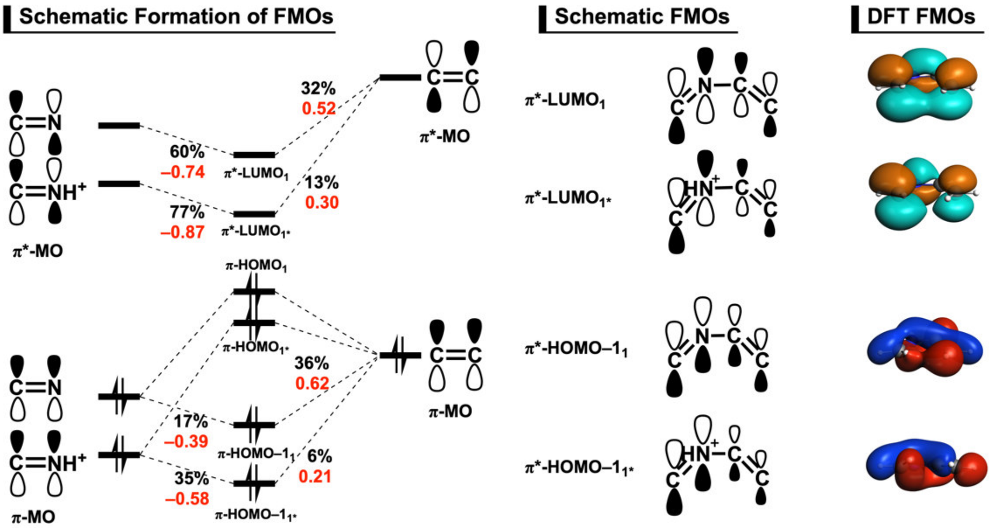

Figure S5. Schematic formation of the frontier molecular orbitals (FMOs) of **1** and **1***, including the contributions (blue) and coefficients (red) of the constituting fragment MOs, with schematic and DFT-computed plots of the FMOs.

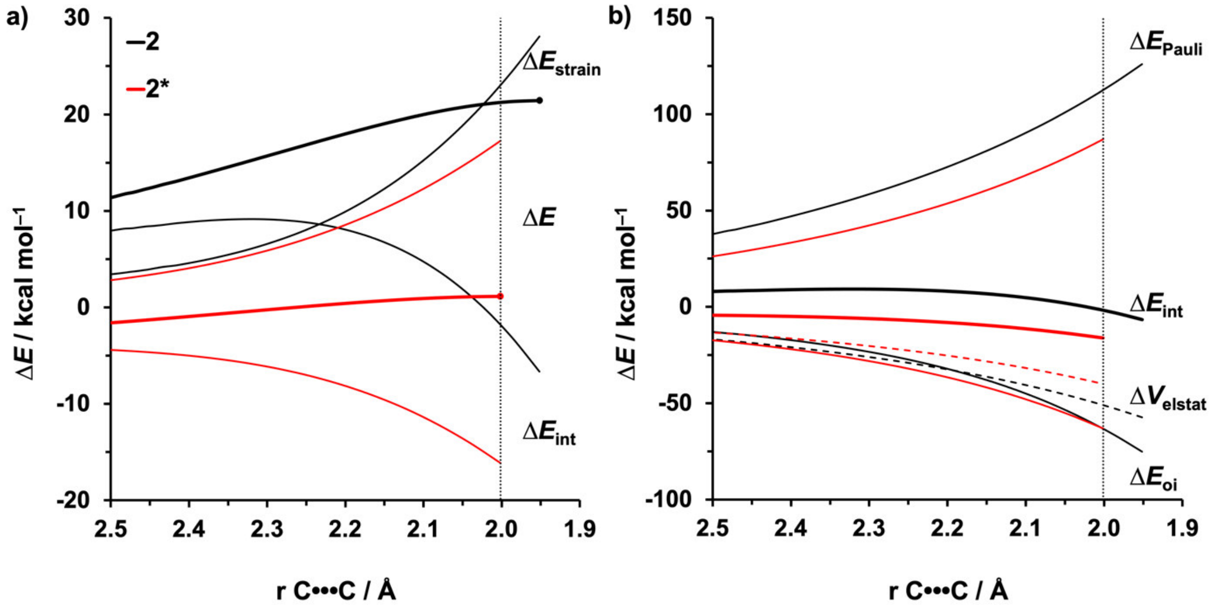

Figure S6. a) Activation strain and b) energy decomposition analyses of aza-Diels-Alder reactions of **2** and **2*** with ethylene along the IRC projected onto the length of the shorter newly forming C•••C bond, computed at BP86/TZ2P. The vertical dotted line indicates the consistent point where the distance of the shorter forming bond is 2.00 Å.

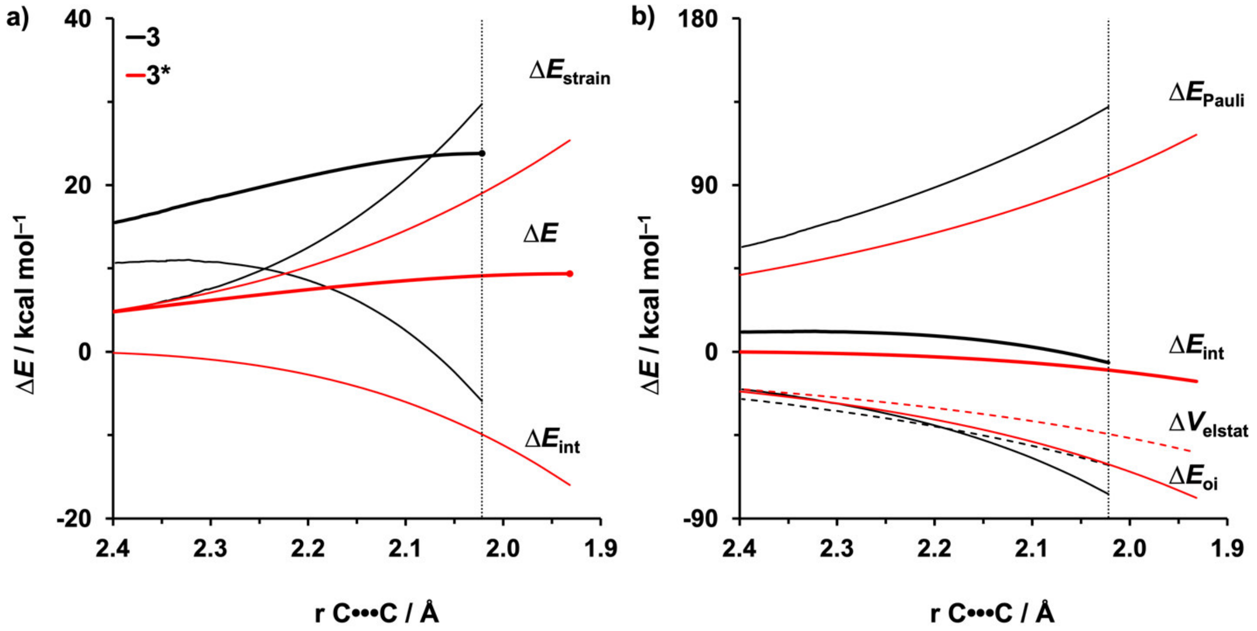

Figure S7. a) Activation strain and b) energy decomposition analyses of aza-Diels-Alder reactions of **3** and **3*** with ethylene along the IRC projected onto the length of the shorter newly forming C•••C bond, computed at BP86/TZ2P. The vertical dotted line indicates the consistent point where the distance of the shorter forming bond is 2.02 Å.

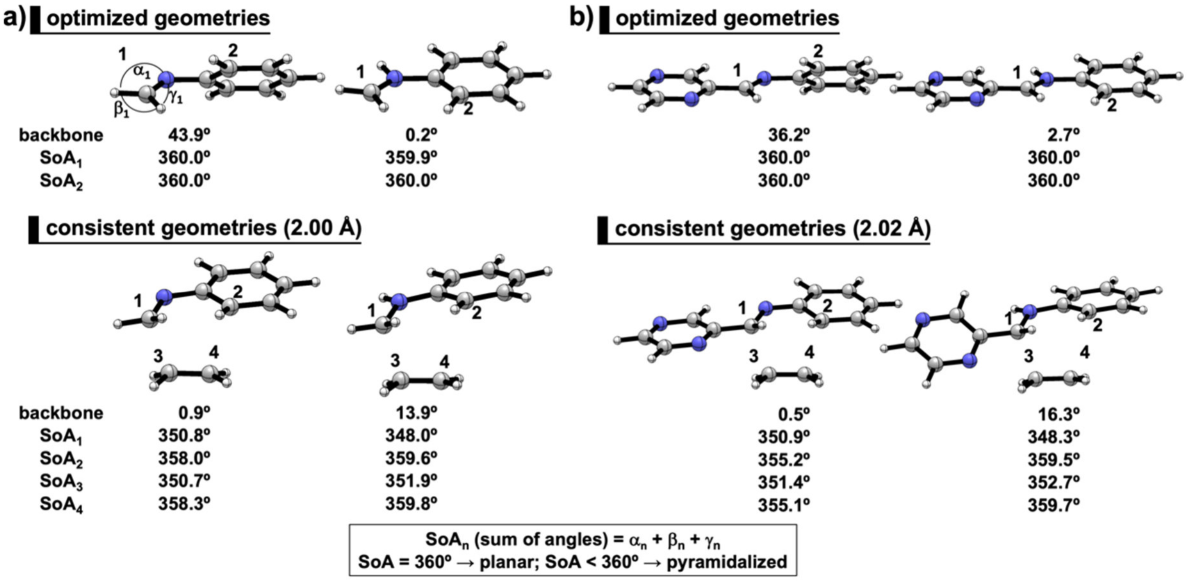

Figure S8. a) Optimized and consistent (2.00 Å) geometries of **2** and **2***, and b) optimized and consistent (2.02 Å) geometries of **3** and **3***, including the geometric information within structures. All were computed at BP86/TZ2P.

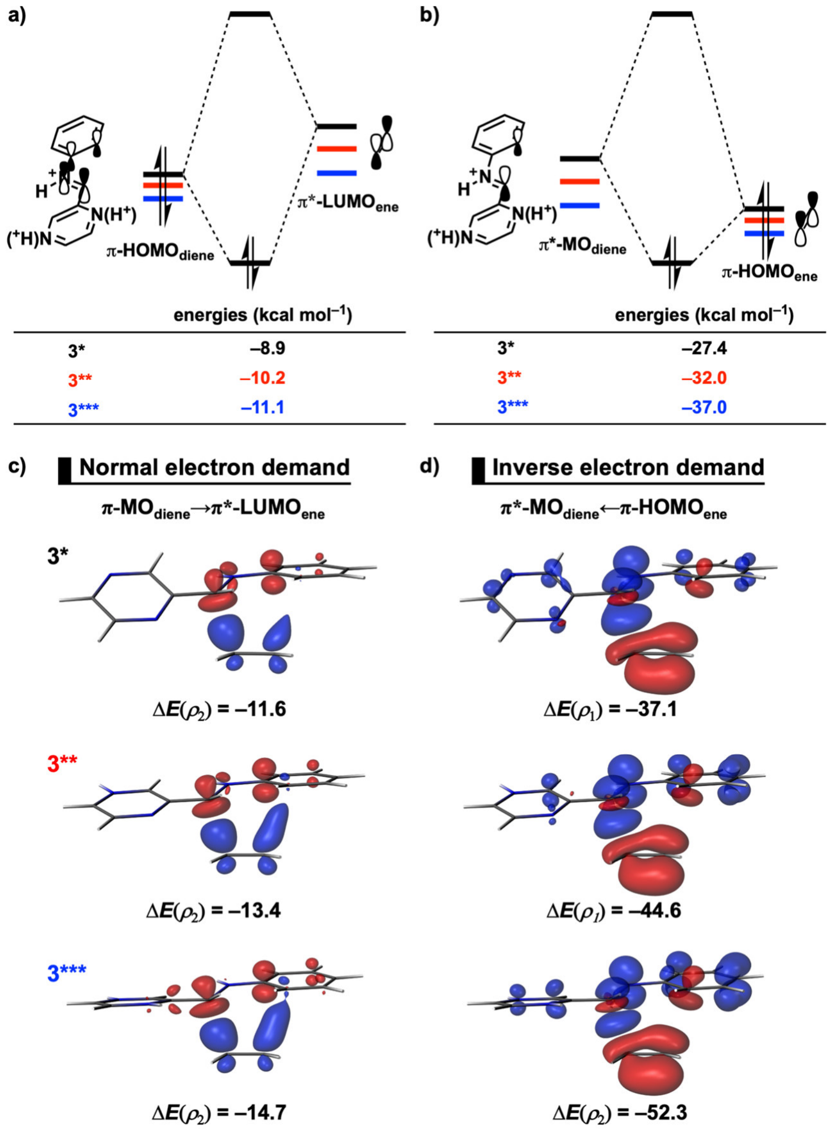

Figure S9. Computed energies for the a) normal electron demand (NED) interactions between the filled orbitals of diene with the π*-LUMO$_{ene}$ and b) inverse electron demand (IED) interactions between the π*-MO$_{diene}$ with the filled orbitals of ethylene, for the aza-Diels-Alder reactions of **3***, **3****, and **3*****. The NED energies were obtained by deleting all virtual orbitals but the π*-LUMO$_{ene}$, and the IED energies were obtained by deleting all virtual orbitals but the interacting π*-MO$_{diene}$. NOCV deformation densities $\Delta\rho$ (isovalue = 0.002 au; electron flow: red → blue) and associated energies $\Delta E(\rho)$ (in kcal mol^{-1}) for the c) NED and d) IED interactions of the aza-Diels-Alder reactions of **3***, **3****, and **3*****. All were computed at the consistent geometries (2.06 Å) at BP86/TZ2P.

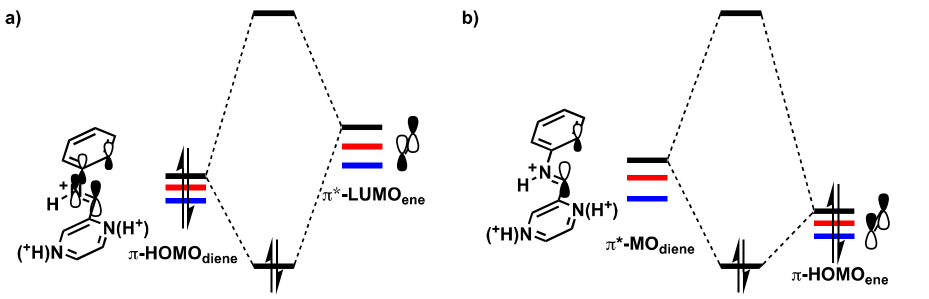

a)

	επ-HOMOdiene (eV)	επ*-LUMOene (eV)	Δε (eV)
3*	–10.4	–7.8	2.6
3**	–13.5	–11.0	2.5
3***	–17.2	–14.8	2.4

b)

		επ*-MOdiene (eV)	επ-HOMOene (eV)	Δε (eV)
3*	π*-LUMO	–9.0	–12.3	3.3
	π*-LUMO+1	–6.4		5.9
	π*-LUMO+2	–5.7		6.6
3**	π*-LUMO	–12.4	–15.4	3.0
	π*-LUMO+1	–11.7		3.7
	π*-LUMO+2	–10.2		5.2
3***	π*-LUMO	–16.3	–19.1	2.8
	π*-LUMO+1	–16.1		3.0
	π*-LUMO+1	–14.5		4.6

Figure S10. Schematic diagrams of the a) normal electron demand (NED) interactions between the π-HOMO$_{diene}$ and π*-LUMO$_{ene}$ and b) inverse electron demand (IED) interactions between the π*-MO$_{diene}$ and π-HOMO$_{ene}$, with the computed orbital energies ε (in eV) and energy gaps Δε (in eV), for the aza-Diels-Alder reactions of **3***, **3****, and **3*****. All data were computed at the consistent geometries (2.06 Å) at BP86/TZ2P.

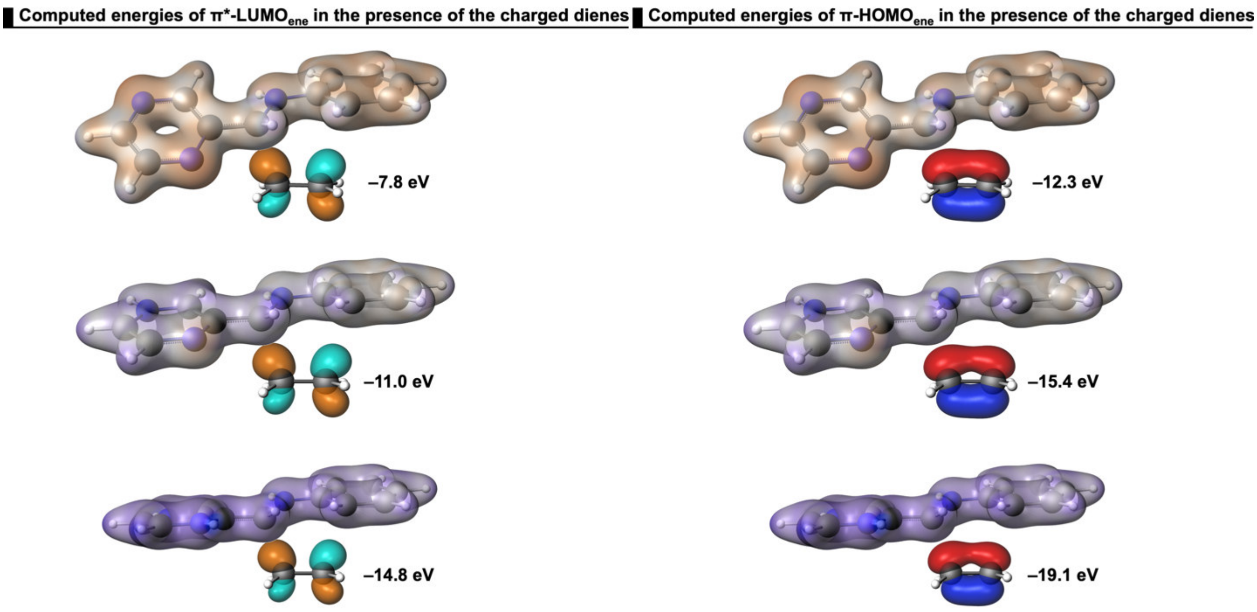

Figure S11. Electrostatic potential maps (at 0.06 Bohr^{-3}) from 0 (red) to +1 (blue) Hartree^{-1} of the fragmental **3***, **3****, and **3*****, and the DFT-computed plots of the MOs (isovalue = 0.09 Bohr$^{-3/2}$) and energies of ethylene in proximity to the fragmental **3***, **3****, and **3*****. All data were computed in the consistent geometries (2.06 Å) at BP86/TZ2P.

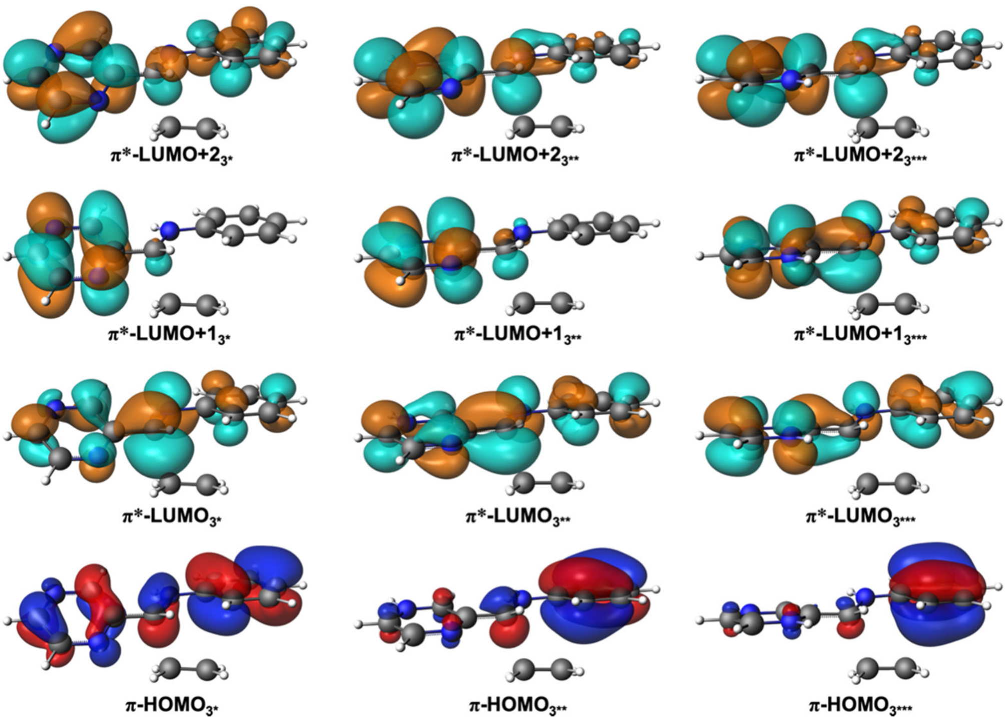

Figure S12. DFT-computed plots of the MOs (isovalue = 0.03 Bohr$^{-3/2}$) of the diene in the aza-Diels-Alder reactions of **3***, **3****, and **3***** at the consistent geometries (2.06 Å), computed at BP86/TZ2P.

Table S2. Electronic energies (kcal mol^{-1}), enthalpies (kcal mol^{-1}), Gibbs free energies (kcal mol^{-1}), the number and wavelength of the imaginary vibrational frequencies and cartesian coordinates (Å), for all stationary points, computed at BP86/TZ2P using ADF2017.107.

---

ethylene
E = -729.1
H = -695.6
G = -711.2
N_{imag} = 0

H	-0.927879000000	1.238707000000	0.000000000000
H	0.927879000000	1.238707000000	0.000000000000
C	0.000000000000	-0.665741000000	0.000000000000
C	0.000000000000	0.665741000000	0.000000000000
H	0.927879000000	-1.238707000000	0.000000000000
H	-0.927879000000	-1.238707000000	0.000000000000

1
E = -1188.4
H = -1140.5
G = -1160.5
N_{imag} = 0

N	-0.125053330000	-0.850428330000	0.740965670000
C	0.360174670000	-1.555807330000	-0.200575330000
C	-0.615178330000	0.432666670000	0.484218670000
C	0.078845670000	1.423862670000	-0.094552330000
H	0.352020670000	-1.231242330000	-1.255565330000
H	0.800808670000	-2.529518330000	0.036530670000
H	-1.608040330000	0.614332670000	0.904300670000
H	1.102057670000	1.274031670000	-0.438158330000
H	-0.345635330000	2.422102670000	-0.177164330000

1–TS
E = -1903.5
H = -1821.1
G = -1844.4
N_{imag} = 1, v = i390.3 cm^{-1}

N	-1.009335000000	-0.773157000000	-0.724736000000
C	1.701170000000	-0.538983000000	0.103158000000
C	-0.368209000000	-1.382660000000	0.248950000000
C	-1.213263000000	0.565413000000	-0.636178000000
C	-0.705889000000	1.385263000000	0.344413000000
C	1.607539000000	0.834942000000	0.103870000000
H	1.803403000000	1.406537000000	1.009264000000
H	1.845243000000	-1.074214000000	-0.832557000000
H	2.004512000000	-1.065105000000	1.007818000000
H	-0.392861000000	-1.036933000000	1.292650000000
H	-0.129361000000	-2.439722000000	0.108239000000
H	1.633547000000	1.396006000000	-0.826569000000

H	-1.683735000000	1.012497000000	-1.516652000000
H	-0.472465000000	1.017042000000	1.339336000000
H	-0.804626000000	2.465912000000	0.248982000000

1*
E = -1114.4
H = -1057.7
G = -1077.9
$\boldsymbol{N}_{\mathrm{imag}} = 0$

C	-3.987473000000	-0.799798000000	0.101235000000
N	-3.511026000000	0.396764000000	0.138786000000
C	-2.197935000000	0.836911000000	-0.213730000000
C	-1.112899000000	0.076315000000	-0.072788000000
H	-4.146703000000	1.144629000000	0.441005000000
H	-5.008146000000	-0.976095000000	0.437967000000
H	-3.367381000000	-1.615946000000	-0.268839000000
H	-2.178549000000	1.867629000000	-0.562972000000
H	-1.134157000000	-0.923367000000	0.360858000000
H	-0.143498000000	0.470920000000	-0.370911000000

1*–TS
E = -1849.0
H = -1757.8
G = -1782.1
$\boldsymbol{N}_{\mathrm{imag}} = 1$, v = i153.5 cm^{-1}

C	-1.257960810000	0.483740870000	-0.534688370000
N	-0.958991810000	-0.752881130000	-0.904953380000
C	0.322786190000	-1.229757120000	-1.185643370000
C	1.422036190000	-0.456217130000	-1.204588370000
H	-1.681453810000	-1.464887120000	-0.793375380000
H	-2.299440810000	0.700178880000	-0.310729370000
H	-0.616476810000	1.294178880000	-0.861496370000
H	0.365004190000	-2.306118120000	-1.338184370000
H	1.399233190000	0.629109880000	-1.146909370000
H	2.392689190000	-0.918516130000	-1.369008370000
C	0.805421190000	0.522951880000	1.503694630000
C	-0.530555810000	0.784462880000	1.573014630000
H	-1.213938810000	0.044200880000	1.989480620000
H	1.211798190000	-0.456744130000	1.750154620000
H	1.526867190000	1.310722880000	1.285845630000
H	-0.887016810000	1.815573880000	1.547386630000

1*–Product
E = -1903.9
H = -1809.4
G = -1831.6
$\boldsymbol{N}_{\mathrm{imag}} = 0$

C	-3.831518000000	-1.052258000000	-0.102261000000
N	-3.372811000000	0.229921000000	0.488361000000
C	-2.187212000000	0.726655000000	0.362516000000

C	-1.098909000000	0.047114000000	-0.364686000000
H	-4.064798000000	0.760220000000	1.023398000000
H	-4.856525000000	-0.884248000000	-0.455521000000
H	-3.878864000000	-1.781615000000	0.720047000000
H	-2.013278000000	1.700053000000	0.828939000000
H	-0.193147000000	0.112096000000	0.261748000000
H	-0.860372000000	0.697237000000	-1.229357000000
C	-2.896146000000	-1.498443000000	-1.220676000000
C	-1.429881000000	-1.389968000000	-0.786065000000
H	-3.075957000000	-0.882108000000	-2.113738000000
H	-3.149448000000	-2.530261000000	-1.490996000000
H	-1.235682000000	-2.070183000000	0.055238000000
H	-0.762576000000	-1.697458000000	-1.598458000000

2
E = -2186.0
H = -2107.4
G = -2131.4
N_{imag} = 0

C	-0.232911000000	-0.623510200000	-0.180413670000
C	-1.354875000000	0.204276800000	-0.354528670000
C	-1.362569000000	1.494104800000	0.171435330000
C	-0.271784000000	1.963085800000	0.911100330000
C	0.831134000000	1.130984800000	1.117752330000
C	0.857243000000	-0.153451200000	0.573639330000
H	-2.205878000000	-0.181508200000	-0.915436670000
H	-2.231614000000	2.134048800000	0.015891330000
H	-0.289414000000	2.965581800000	1.338483330000
H	1.675015000000	1.480775800000	1.713476330000
H	1.705624000000	-0.813108200000	0.758837330000
N	-0.276610000000	-1.924103200000	-0.724011670000
C	0.753186000000	-2.405810200000	-1.299140670000
H	1.687121000000	-1.837271200000	-1.453881670000
H	0.716332000000	-3.434096200000	-1.673202670000

2–TS
E = -2893.7
H = -2780.6
G = -2807.5
N_{imag} = 1, v = i454.6 cm^{-1}

C	-0.642927140000	0.500469100000	-0.615641140000
C	-1.876153140000	-0.120026900000	-0.982564140000
C	-1.911177140000	-1.424012900000	-1.422271140000
C	-0.717615140000	-2.183010900000	-1.521842140000
C	0.494756860000	-1.611791900000	-1.189708140000
C	0.555487860000	-0.280551900000	-0.711836140000
H	-2.783018140000	0.476832100000	-0.883489140000
H	-2.863272140000	-1.881715900000	-1.693370140000
H	-0.758748140000	-3.211311900000	-1.882061140000
H	1.417919860000	-2.182039900000	-1.303680140000

H	1.521563860000	0.219778100000	-0.708170140000
N	-0.694392140000	1.736448100000	-0.061535140000
C	0.444423860000	2.228014100000	0.444372860000
H	0.016268860000	1.394905100000	2.584288860000
H	1.795038860000	1.687011100000	2.257412860000
H	0.253295860000	-0.933054900000	1.791423860000
H	2.037703860000	-0.624573900000	1.439145860000
H	1.408562860000	2.069820100000	-0.066480140000
C	1.049423860000	-0.211094900000	1.629949860000
C	0.892593860000	1.132031100000	1.992137860000
H	0.360262860000	3.217877100000	0.903916860000

2–Product
E = -2926.3
H = -2810.7
G = -2837.0
N_{imag} = 0

C	-0.695827000000	-0.562579330000	0.447541570000
C	-1.884972000000	-0.031915330000	1.086563570000
C	-1.926585000000	1.246635670000	1.539475570000
C	-0.788761000000	2.139489670000	1.402561570000
C	0.360143000000	1.722193670000	0.833503570000
C	0.530458000000	0.328721670000	0.315461570000
H	-2.739907000000	-0.703585330000	1.169100570000
H	-2.833803000000	1.628061670000	2.010196570000
H	-0.880036000000	3.162514670000	1.769907570000
H	1.209601000000	2.401876670000	0.733310570000
H	1.331492000000	-0.156532330000	0.912295570000
N	-0.692377000000	-1.777815330000	-0.007981430000
C	0.548476000000	-2.164804330000	-0.658501430000
H	0.237634000000	-1.189104330000	-2.550019430000
H	1.940963000000	-1.444241330000	-2.159856430000
H	0.420304000000	0.974126670000	-1.757215430000
H	2.064845000000	0.686672670000	-1.186967430000
H	1.357031000000	-2.244232330000	0.097720570000
C	1.040264000000	0.290636670000	-1.162737430000
C	0.973270000000	-1.142932330000	-1.735923430000
H	0.427787000000	-3.163187330000	-1.098436430000

2*
E = -2120.3
H = -2032.9
G = -2057.4
N_{imag} = 0

C	-5.368143000000	-1.035270000000	2.739153000000
C	-6.566677000000	-1.404530000000	3.369436000000
C	-6.709338000000	-2.701999000000	3.850350000000
C	-5.662994000000	-3.615317000000	3.700571000000
C	-4.469653000000	-3.234421000000	3.069050000000
C	-4.310552000000	-1.943413000000	2.583057000000

H	-7.378315000000	-0.682882000000	3.481324000000
H	-7.635030000000	-2.998544000000	4.340686000000
H	-5.772788000000	-4.631834000000	4.076675000000
H	-3.659144000000	-3.952904000000	2.956876000000
H	-3.378477000000	-1.662146000000	2.095420000000
N	-5.290222000000	0.302357000000	2.272270000000
C	-4.336052000000	0.925504000000	1.660487000000
H	-3.405513000000	0.413776000000	1.426520000000
H	-6.140982000000	0.844544000000	2.461668000000
H	-4.483574000000	1.967507000000	1.386269000000

2*–TS
E = -2848.3
H = -2726.3
G = -2754.4
N_{imag} = 1, v = i269.7 cm^{-1}

C	-0.917520230000	-0.084881550000	0.020849860000
C	-1.804252230000	0.835329450000	-0.576342140000
C	-1.920628230000	2.110568450000	-0.049655140000
C	-1.162110230000	2.488286450000	1.072068860000
C	-0.290828230000	1.580978450000	1.667016860000
C	-0.157968230000	0.287421450000	1.151488860000
H	-2.388266230000	0.540942450000	-1.449870140000
H	-2.603849230000	2.823065450000	-0.509937140000
H	-1.270134230000	3.490313450000	1.485248860000
H	0.276829770000	1.864436450000	2.552105860000
H	0.442711770000	-0.442245550000	1.690577860000
N	-0.777447230000	-1.344838550000	-0.556838140000
C	0.232545770000	-2.198757550000	-0.299985140000
H	2.529298770000	-2.332640550000	-0.694931140000
H	1.879798770000	0.681807450000	-0.912529140000
H	2.704032770000	-0.177272550000	0.508612860000
H	0.569263770000	-2.267125550000	0.730517860000
H	-1.305308230000	-1.490180550000	-1.417649140000
C	2.166878770000	-0.254202550000	-0.436335140000
C	1.941461770000	-1.477143550000	-1.036658140000
H	1.664599770000	-1.493806550000	-2.091955140000
H	0.190890770000	-3.140054550000	-0.845802140000

2*–Product
E = -2882.0
H = -2757.5
G = -2784.2
N_{imag} = 0

C	-0.879019500000	0.199229950000	0.120081770000
C	-2.003754500000	-0.623845050000	0.377233770000
C	-2.005854500000	-1.916748050000	-0.081541230000
C	-0.907289500000	-2.481490050000	-0.816646230000
C	0.184388500000	-1.730779050000	-1.090822230000
C	0.295332500000	-0.312461050000	-0.654353230000

H	-2.847846500000	-0.229421050000	0.943783770000
H	-2.872697500000	-2.545920050000	0.124225770000
H	-0.970114500000	-3.517040050000	-1.147319230000
H	1.025371500000	-2.141426050000	-1.650609230000
H	0.340899500000	0.303105950000	-1.578281230000
N	-0.790903500000	1.450987950000	0.547183770000
C	0.423350500000	2.235509950000	0.322301770000
H	2.458148500000	-0.135391050000	-0.615078230000
H	1.773544500000	-0.770210050000	0.881575770000
H	0.473827500000	2.506510950000	-0.745641230000
H	-1.561429500000	1.856046950000	1.076704770000
C	1.642762500000	-0.007361050000	0.105858770000
C	1.652512500000	1.406188950000	0.730543770000
H	1.670584500000	1.332696950000	1.824905770000
H	2.551060500000	1.957874950000	0.429652770000
H	0.347126500000	3.163939950000	0.896239770000

3
E = -3537.5
H = -3421.3
G = -3453.0
$\boldsymbol{N}_{\mathrm{imag}}$ = 0

C	-5.470219000000	-0.959151000000	2.645072000000
C	-6.460202000000	-1.292866000000	3.586736000000
C	-6.470822000000	-2.551978000000	4.180146000000
C	-5.520619000000	-3.510879000000	3.816504000000
C	-4.556351000000	-3.199107000000	2.854200000000
C	-4.523898000000	-1.933562000000	2.272661000000
H	-7.203647000000	-0.539872000000	3.846329000000
H	-7.233216000000	-2.792918000000	4.920992000000
H	-5.542464000000	-4.502650000000	4.267481000000
H	-3.829243000000	-3.951343000000	2.547299000000
H	-3.789866000000	-1.705139000000	1.499927000000
N	-5.516165000000	0.325457000000	2.083078000000
C	-4.431963000000	0.934936000000	1.769014000000
C	-4.444325000000	2.252383000000	1.120823000000
C	-5.636769000000	2.931132000000	0.799996000000
N	-5.636800000000	4.130030000000	0.214566000000
C	-4.431825000000	4.656599000000	-0.056390000000
C	-3.245280000000	3.989499000000	0.256677000000
N	-3.239170000000	2.789528000000	0.845108000000
H	-6.600028000000	2.474578000000	1.032879000000
H	-3.423731000000	0.534951000000	1.973335000000
H	-4.415228000000	5.637392000000	-0.536928000000
H	-2.274034000000	4.434661000000	0.027410000000

3–TS
E = -4242.8
H = -4092.2
G = -4126.4

N_{imag} = 1, v = i497.2 cm^{-1}

C	-0.774097660000	-0.897454070000	-1.108714170000
C	-2.012022660000	-1.520646070000	-1.458238170000
C	-2.045011660000	-2.809767070000	-1.932223170000
C	-0.843494660000	-3.551973070000	-2.093958170000
C	0.369410340000	-2.983248070000	-1.781582170000
C	0.435685340000	-1.667393070000	-1.241236170000
H	-2.921717660000	-0.936418070000	-1.320580170000
H	-2.998656660000	-3.271226070000	-2.189307170000
H	-0.886723660000	-4.566193070000	-2.491196170000
H	1.293936340000	-3.538287070000	-1.945404170000
H	1.382111340000	-1.136053070000	-1.330744170000
N	-0.813357660000	0.317025930000	-0.527526170000
C	0.336649340000	0.812860930000	-0.047306170000
C	0.295743340000	2.138142930000	0.622313830000
C	-0.911175660000	2.785254930000	0.943780830000
N	-0.942107660000	3.974536930000	1.552881830000
C	0.246604340000	4.524564930000	1.842065830000
C	1.448407340000	3.891933930000	1.520084830000
N	1.482999340000	2.699785930000	0.914421830000
H	-1.861096660000	2.318006930000	0.682189830000
H	1.936720340000	-2.197908070000	0.685845830000
H	0.238105340000	5.495264930000	2.343033830000
H	2.408960340000	4.356670930000	1.756229830000
H	0.172656340000	-2.489463070000	1.113115830000
H	1.294883340000	0.651330930000	-0.566667170000
C	0.961463340000	-1.769511070000	0.908862830000
C	0.874860340000	-0.466685070000	1.422650830000
H	0.032740340000	-0.228293070000	2.071189830000
H	1.797525340000	0.065138930000	1.656015830000

3–Product

E = -4271.6

H = -4118.8

G = -4152.6

N_{imag} = 0

C	-0.821735340000	0.897727790000	0.973879590000
C	-2.025831340000	1.452452790000	1.558868590000
C	-2.049821340000	2.722311790000	2.036230590000
C	-0.879104340000	3.579060790000	1.982291590000
C	0.284898660000	3.134724790000	1.468281590000
C	0.436687660000	1.747676790000	0.929998590000
H	-2.905815340000	0.809691790000	1.581375590000
H	-2.968937340000	3.123879790000	2.464448590000
H	-0.957588340000	4.596030790000	2.367667590000
H	1.161104660000	3.785327790000	1.431386590000
H	1.185545660000	1.224234790000	1.560331590000
N	-0.832982340000	-0.304488210000	0.485915590000
C	0.422301660000	-0.727207210000	-0.103645410000
C	0.323536660000	-2.106328210000	-0.721306410000

C	-0.903553340000	-2.735505210000	-0.980274410000
N	-0.979922340000	-3.944994210000	-1.551333410000
C	0.184784660000	-4.529250210000	-1.860718410000
C	1.408970660000	-3.911399210000	-1.600841410000
N	1.486489660000	-2.701866210000	-1.034086410000
H	-1.835116340000	-2.241933210000	-0.704351410000
H	2.077660660000	2.046707790000	-0.467498410000
H	0.140080660000	-5.516881210000	-2.326026410000
H	2.352262660000	-4.403036210000	-1.852340410000
H	0.492486660000	2.459021790000	-1.125443410000
H	1.207283660000	-0.791256210000	0.679375590000
C	1.030500660000	1.720105790000	-0.518360410000
C	0.912389660000	0.316782790000	-1.147233410000
H	0.183379660000	0.323165790000	-1.969015410000
H	1.870043660000	-0.024758210000	-1.557576410000

3*
E = -3474.2
H = -3349.6
G = -3382.1
$\boldsymbol{N}_{\mathrm{imag}}$ = 0

C	-5.411043000000	-1.011041000000	2.719022000000
C	-6.624245000000	-1.434760000000	3.282237000000
C	-6.715458000000	-2.709925000000	3.829950000000
C	-5.602725000000	-3.553153000000	3.816049000000
C	-4.394794000000	-3.119437000000	3.254423000000
C	-4.286985000000	-1.848827000000	2.702953000000
H	-7.490901000000	-0.771198000000	3.288556000000
H	-7.654880000000	-3.043755000000	4.266877000000
H	-5.671976000000	-4.551651000000	4.245254000000
H	-3.528475000000	-3.778897000000	3.249496000000
H	-3.338295000000	-1.527970000000	2.276787000000
N	-5.390750000000	0.298254000000	2.174359000000
C	-4.423756000000	0.924448000000	1.554088000000
C	-4.483757000000	2.268386000000	1.031187000000
C	-5.590326000000	3.135269000000	1.125237000000
N	-5.565870000000	4.362581000000	0.608570000000
C	-4.433166000000	4.731689000000	-0.002607000000
C	-3.319333000000	3.875585000000	-0.095337000000
N	-3.337890000000	2.650008000000	0.415438000000
H	-6.521366000000	2.850688000000	1.626861000000
H	-3.479875000000	0.399271000000	1.406416000000
H	-4.407276000000	5.735815000000	-0.430480000000
H	-2.402078000000	4.195217000000	-0.593177000000
H	-6.275320000000	0.799259000000	2.295869000000

3*–TS
E = -4193.9
H = -4034.9
G = -4070.0

N_{imag} = 1, ν = i305.4 cm^{-1}

C	1.433024730000	-0.790429330000	0.325992330000
C	2.698323730000	-0.982469330000	-0.270385670000
C	3.359011730000	-2.184997330000	-0.099764670000
C	2.780937730000	-3.217512330000	0.663220330000
C	1.540428730000	-3.034111330000	1.256881330000
C	0.848219730000	-1.825302330000	1.093010330000
H	3.145028730000	-0.184950330000	-0.865764670000
H	4.334088730000	-2.332648330000	-0.561769670000
H	3.319025730000	-4.154228330000	0.800580330000
H	1.103871730000	-3.818248330000	1.873406330000
H	-0.048099270000	-1.644675330000	1.682057330000
N	0.759176730000	0.399752670000	0.100515330000
C	-0.588100270000	0.548760670000	0.277369330000
C	-1.180676270000	1.899682670000	0.054899330000
N	-2.522994270000	1.955155670000	0.073780330000
C	-3.086515270000	3.150578670000	-0.088875670000
C	-2.311986270000	4.300409670000	-0.312569670000
N	-0.979099270000	4.255542670000	-0.298748670000
C	-0.412076270000	3.063592670000	-0.093726670000
H	-1.622724270000	-2.533576330000	0.010377330000
H	-4.176449270000	3.197366670000	-0.058298670000
H	-2.779112270000	5.267622670000	-0.506608670000
H	-0.377130270000	-2.462368330000	-1.357491670000
H	0.677670730000	3.057969670000	-0.003093670000
H	-0.983174270000	0.055492670000	1.166068330000
H	1.209831730000	1.032293670000	-0.560861670000
C	-1.099105270000	-1.937227330000	-0.735413670000
C	-1.464770270000	-0.619931330000	-0.986382670000
H	-1.120390270000	-0.171968330000	-1.920541670000
H	-2.456237270000	-0.289576330000	-0.657860670000

3*–Product

E = -4223.2

H = -4062.4

G = -4094.9

N_{imag} = 0

C	1.349360070000	0.723447900000	-0.388627700000
C	2.765023070000	0.787626900000	-0.421925700000
C	3.378110070000	2.013361900000	-0.464727700000
C	2.643576070000	3.248628900000	-0.475964700000
C	1.291514070000	3.235503900000	-0.454228700000
C	0.516555070000	1.965672900000	-0.424948700000
H	3.351181070000	-0.131196100000	-0.400173700000
H	4.467572070000	2.055478900000	-0.483791700000
H	3.192222070000	4.188531900000	-0.502806700000
H	0.718236070000	4.162795900000	-0.465723700000
H	-0.051422930000	1.917595900000	-1.378991700000
N	0.684238070000	-0.420123100000	-0.323222700000
C	-0.783935930000	-0.448303100000	-0.245094700000

C	-1.299132930000	-1.855954100000	-0.030807700000
N	-2.266521930000	-2.252441100000	-0.871434700000
C	-2.753347930000	-3.483264100000	-0.683772700000
C	-2.265387930000	-4.322114100000	0.326329300000
N	-1.302353930000	-3.928762100000	1.166272300000
C	-0.829559930000	-2.689409100000	0.990680300000
H	-1.322949930000	2.703754900000	0.455217300000
H	-3.546598930000	-3.810998100000	-1.358128700000
H	-2.660907930000	-5.330647100000	0.460840300000
H	-0.105089930000	2.238815900000	1.644303300000
H	-0.057511930000	-2.361341100000	1.694224300000
H	-1.182967930000	-0.116434100000	-1.219048700000
H	1.191809070000	-1.305151100000	-0.331036700000
C	-0.580245930000	1.937667900000	0.703994300000
C	-1.233122930000	0.546473900000	0.852685300000
H	-0.982644930000	0.111952900000	1.828561300000
H	-2.325692930000	0.618827900000	0.801349300000

3**
E = -3325.9
H = -3193.1
G = -3225.3
$\boldsymbol{N}_{\mathrm{imag}}$ = 0

C	-5.421093000000	-1.026677000000	2.724704000000
C	-6.629288000000	-1.463158000000	3.317853000000
C	-6.696779000000	-2.732992000000	3.866350000000
C	-5.568917000000	-3.563922000000	3.826605000000
C	-4.368162000000	-3.122726000000	3.236061000000
C	-4.281319000000	-1.858257000000	2.682274000000
H	-7.501823000000	-0.806870000000	3.342405000000
H	-7.620858000000	-3.080946000000	4.324726000000
H	-5.618763000000	-4.563583000000	4.257919000000
H	-3.500840000000	-3.780848000000	3.215666000000
H	-3.345276000000	-1.532821000000	2.232001000000
N	-5.430604000000	0.268943000000	2.188725000000
C	-4.475371000000	0.926633000000	1.572822000000
C	-4.543446000000	2.276323000000	1.038158000000
C	-5.644208000000	3.148746000000	1.091898000000
N	-5.500814000000	4.366388000000	0.533760000000
C	-4.365988000000	4.778202000000	-0.064755000000
C	-3.296027000000	3.873850000000	-0.092886000000
N	-3.386723000000	2.663035000000	0.443304000000
H	-6.612085000000	2.941307000000	1.544684000000
H	-3.515132000000	0.432732000000	1.429846000000
H	-4.344249000000	5.781303000000	-0.488017000000
H	-2.350897000000	4.151471000000	-0.563492000000
H	-6.336546000000	0.732105000000	2.317054000000
H	-6.296882000000	5.013532000000	0.566503000000

3–TS**

E = -4053.8
H = -3886.5
G = -3921.8
N_{imag} = 1, v = i298.6 cm^{-1}

C	0.114722530000	-1.764906760000	0.340687010000
C	-0.667776500000	-2.777564230000	0.941778990000
C	-0.040153630000	-3.839298880000	1.562440420000
C	1.368889490000	-3.920212230000	1.592522500000
C	2.144593030000	-2.932245280000	0.998911140000
C	1.533937000000	-1.840340380000	0.370213540000
H	-1.756982640000	-2.714850270000	0.920822430000
H	-0.637347770000	-4.620097290000	2.031413750000
H	1.847696040000	-4.775620010000	2.067793340000
H	3.230330840000	-3.013863730000	0.993834180000
H	2.145853220000	-1.166230310000	-0.225593600000
N	-0.533005220000	-0.684194300000	-0.240615700000
C	0.083427740000	0.491717520000	-0.522981380000
C	-0.641151020000	1.601779200000	-1.185213260000
N	0.148648810000	2.638518380000	-1.540543440000
C	-0.378547530000	3.688677730000	-2.163617430000
C	-1.741483500000	3.758816550000	-2.470313910000
N	-2.502738750000	2.710703940000	-2.106149430000
C	-2.011598040000	1.625319760000	-1.471145210000
H	2.577118290000	0.264242380000	1.757829290000
H	0.291213270000	4.505093570000	-2.439344830000
H	-2.222117570000	4.590739100000	-2.982252760000
H	1.085852660000	-0.319532540000	2.690666900000
H	-2.727993260000	0.836754880000	-1.252002460000
H	1.104924820000	0.415204370000	-0.888789550000
H	-1.544641480000	-0.693013640000	-0.110112380000
C	1.498820140000	0.308183030000	1.903142500000
C	0.732328350000	1.278099120000	1.271632660000
H	-0.251004520000	1.502645050000	1.688721110000
H	1.251675570000	2.113381780000	0.796933020000
H	-3.503490350000	2.732093490000	-2.330667420000

3–Product**

E = -4088.9
H = -3919.8
G = -3952.6
N_{imag} = 0

C	1.086820810000	1.351898940000	0.125576260000
C	1.862008810000	2.355635940000	-0.483142740000
C	2.939541810000	2.882074940000	0.198840260000
C	3.315319810000	2.450583940000	1.510646260000
C	2.585777810000	1.499461940000	2.142533260000
C	1.373324810000	0.899653940000	1.521434260000
H	1.624170810000	2.701866940000	-1.489384740000
H	3.537405810000	3.654558940000	-0.286865740000
H	4.188375810000	2.894322940000	1.986492260000

H	2.848028810000	1.157378940000	3.144306260000
H	0.521974810000	1.304373940000	2.116946260000
N	0.042329810000	0.777524940000	-0.490174740000
C	-0.761891190000	-0.238496060000	0.194737260000
C	-1.786659190000	-0.896328060000	-0.709780740000
N	-3.011294190000	-1.055551060000	-0.178982740000
C	-3.957103190000	-1.681535060000	-0.881775740000
C	-3.717630190000	-2.171906060000	-2.167403740000
N	-2.480482190000	-2.000296060000	-2.668740740000
C	-1.490891190000	-1.384292060000	-1.986074740000
H	1.136528810000	-0.874916060000	2.743286260000
H	-4.939072190000	-1.798199060000	-0.420049740000
H	-4.456340190000	-2.679526060000	-2.785560740000
H	2.266945810000	-1.080901060000	1.405926260000
H	-0.521546190000	-1.316010060000	-2.476531740000
H	-1.345162190000	0.239851940000	0.999898260000
H	-0.288162190000	1.189487940000	-1.361657740000
C	1.295214810000	-0.656938060000	1.681755260000
C	0.187842810000	-1.297407060000	0.815160260000
H	0.639128810000	-1.873884060000	-0.003989740000
H	-0.416167190000	-1.992634060000	1.410438260000
H	-2.278339190000	-2.359856060000	-3.607860740000

3***
E = -3083.9
H = -2943.6
G = -2975.9
$\boldsymbol{N}_{\mathrm{imag}}$ = 0

C	-5.415756000000	-1.048595000000	2.664620000000
C	-6.667964000000	-1.488467000000	3.220006000000
C	-6.764108000000	-2.749064000000	3.761683000000
C	-5.626391000000	-3.589727000000	3.763767000000
C	-4.383747000000	-3.154983000000	3.215883000000
C	-4.267004000000	-1.899722000000	2.668726000000
H	-7.537078000000	-0.826041000000	3.209829000000
H	-7.704973000000	-3.100105000000	4.185784000000
H	-5.697390000000	-4.591628000000	4.193363000000
H	-3.525493000000	-3.827089000000	3.235494000000
H	-3.309579000000	-1.583810000000	2.257233000000
N	-5.408459000000	0.225895000000	2.140306000000
C	-4.416291000000	0.905140000000	1.550137000000
C	-4.491035000000	2.231069000000	1.037188000000
C	-5.604424000000	3.113312000000	1.057258000000
N	-5.497440000000	4.334036000000	0.529613000000
C	-4.370278000000	4.829906000000	-0.049767000000
C	-3.264054000000	3.995590000000	-0.086564000000
N	-3.351526000000	2.758911000000	0.440510000000
H	-6.571514000000	2.859003000000	1.488644000000
H	-6.326924000000	4.948748000000	0.562643000000
H	-4.384517000000	5.843274000000	-0.452597000000

H	-2.312799000000	4.299267000000	-0.529259000000
H	-2.507082000000	2.174105000000	0.390542000000
H	-3.465015000000	0.381781000000	1.457962000000
H	-6.322219000000	0.685628000000	2.234242000000

3*–TS**

E = -3815.4

H = -3640.8

G = -3676.5

$\boldsymbol{N}_{\mathrm{imag}}$ = 1, v = i336.0 cm^{-1}

C	1.218334030000	1.408956500000	0.152402470000
C	2.245459030000	2.070102500000	-0.584995530000
C	3.248293030000	2.719586500000	0.094847470000
C	3.254061030000	2.749076500000	1.518013470000
C	2.249854030000	2.111575500000	2.253168470000
C	1.221414030000	1.436458500000	1.592182470000
H	2.242780030000	2.043816500000	-1.676221530000
H	4.046249030000	3.218013500000	-0.456181530000
H	4.035542030000	3.305308500000	2.038108470000
H	2.241174030000	2.181206500000	3.340643470000
H	0.334514030000	1.159313500000	2.159596470000
N	0.253603030000	0.724849500000	-0.537953530000
C	-0.629469970000	-0.158622500000	0.052134470000
C	-1.665005970000	-0.779348500000	-0.772436530000
N	-2.806979970000	-1.239182500000	-0.145152530000
C	-3.828329970000	-1.866653500000	-0.775862530000
C	-3.752992970000	-2.069983500000	-2.140714530000
N	-2.634195970000	-1.627446500000	-2.776171530000
C	-1.610794970000	-1.016598500000	-2.156578530000
H	1.357522030000	-0.956516500000	2.753136470000
H	-4.684636970000	-2.184375500000	-0.178353530000
H	-4.533137970000	-2.558612500000	-2.725279530000
H	2.518854030000	-0.952170500000	1.305113470000
H	-0.767167970000	-0.736604500000	-2.784990530000
H	-1.002753970000	0.176943500000	1.020456470000
H	0.374985030000	0.725370500000	-1.551421530000
C	1.503235030000	-1.075200500000	1.679805470000
C	0.513396030000	-1.660955500000	0.882100470000
H	0.834764030000	-2.121533500000	-0.053916530000
H	-0.294624970000	-2.178879500000	1.406468470000
H	-2.569347970000	-1.773079500000	-3.795065530000
H	-2.914593970000	-1.074814500000	0.863117470000

3*–Product**

E = -3852.4

H = -3675.6

G = -3709.7

$\boldsymbol{N}_{\mathrm{imag}}$ = 0

C	-0.309576810000	0.333204840000	1.744265210000
C	-1.000401960000	-0.105314660000	2.886456750000

C	-1.477860230000	0.824785490000	3.806880330000
C	-1.303196710000	2.224244500000	3.639708600000
C	-0.594391130000	2.693755200000	2.565365990000
C	0.054016930000	1.775248190000	1.593546200000
H	-1.194871720000	-1.167710250000	3.043520630000
H	-2.025650080000	0.460882060000	4.678719730000
H	-1.731146640000	2.913416000000	4.368009990000
H	-0.420549040000	3.763697610000	2.432503600000
H	1.100638580000	1.766565410000	1.999210440000
N	0.124060010000	-0.545801550000	0.808925340000
C	0.730089330000	-0.121329800000	-0.457245160000
C	0.600205600000	-1.194630480000	-1.520231860000
N	1.730480240000	-1.691770350000	-2.110678880000
C	1.727090180000	-2.597430900000	-3.122754420000
C	0.512535940000	-3.066269860000	-3.589907070000
N	-0.617393420000	-2.591282750000	-3.006044630000
C	-0.613042360000	-1.688842490000	-1.999293700000
H	2.645688950000	-1.354639750000	-1.782049130000
H	2.685738070000	-2.926894730000	-3.527238390000
H	0.421050950000	-3.794622560000	-4.397275250000
H	-1.525439870000	-2.929974890000	-3.357804290000
H	-1.578079940000	-1.370571030000	-1.607413630000
H	1.806066360000	0.086368110000	-0.309167990000
H	0.101500870000	-1.537089280000	1.050434880000
C	0.175175540000	2.301508020000	0.126237300000
C	0.029561590000	1.188798180000	-0.924762340000
H	-1.033394650000	0.967432180000	-1.097773050000
H	0.461374290000	1.523360510000	-1.876267750000
H	-0.613151230000	3.040761130000	-0.062141310000
H	1.132872370000	2.820147910000	0.004263870000
